# Nanocarriers as Magic Bullets in the Treatment of Leukemia

**DOI:** 10.3390/nano10020276

**Published:** 2020-02-06

**Authors:** Mohammad Houshmand, Francesca Garello, Paola Circosta, Rachele Stefania, Silvio Aime, Giuseppe Saglio, Claudia Giachino

**Affiliations:** 1 Department of Clinical and Biological Sciences, University of Torino, 10043 Torino, Italy; mohammad.houshmand@unito.it (M.H.); paola.circosta@unito.it (P.C.); giuseppe.saglio@unito.it (G.S.); 2 Molecular and Preclinical Imaging Centres, Department of Molecular Biotechnology and Health Sciences, University of Torino, 10126 Torino, Italy; francesca.garello@unito.it (F.G.); rachele.stefania@unito.it (R.S.); silvio.aime@unito.it (S.A.)

**Keywords:** nanocarrier, nanosystem, nanoparticle, liposome, leukemia, AML, ALL, CLL, CML, targeted therapy

## Abstract

Leukemia is a type of hematopoietic stem/progenitor cell malignancy characterized by the accumulation of immature cells in the blood and bone marrow. Treatment strategies mainly rely on the administration of chemotherapeutic agents, which, unfortunately, are known for their high toxicity and side effects. The concept of targeted therapy as magic bullet was introduced by Paul Erlich about 100 years ago, to inspire new therapies able to tackle the disadvantages of chemotherapeutic agents. Currently, nanoparticles are considered viable options in the treatment of different types of cancer, including leukemia. The main advantages associated with the use of these nanocarriers summarized as follows: i) they may be designed to target leukemic cells selectively; ii) they invariably enhance bioavailability and blood circulation half-life; iii) their mode of action is expected to reduce side effects. FDA approval of many nanocarriers for treatment of relapsed or refractory leukemia and the desired results extend their application in clinics. In the present review, different types of nanocarriers, their capability in targeting leukemic cells, and the latest preclinical and clinical data are discussed.

## 1. Introduction

Leukemia is a heterogeneous disease resulting from the transformation of hematopoietic stem/progenitor cells and it is the 10th most prevalent cause of cancer in the world [1,2]. Leukemia is divided into the lymphoid and myeloid lineages, and the acute or chronic phase is determined by the maturity stage of the cells [3]. For many years, chemotherapeutic agents have been considered as the choice for the treatment of leukemia but their efficacy, due to the appearance and development of chemoresistance and unwanted side effects, are compromised [4]. Furthermore, low solubility and bioavailability of some drugs and a need to increase the selectivity of the treatment implied marked modifications of treatment strategies. It has been shown that, by using conventional treatment, one can eradicate the bulk disease population whereas some resistant cells and also leukemic stem cells (LSCs) persist even after the treatment [5,6]. To tackle these problems and due to the increase in our knowledge about the pathogenesis of leukemia, many targeting-based therapies, ranging from antibodies to inhibitors of signaling pathways, have been introduced [7,8]. Simultaneously, the outstanding development in the field of nanomaterials for cancer treatment pave the way to new therapeutic routs aimed at pursuing targeted therapy, reduction of toxicity, controlled release, delivery of therapeutic RNAs, and a desirable carrier for combinatorial treatment [9]. All of these features led to the Food and Drug Administration (FDA) approval of many nanoparticles for the treatment of relapsed or refractory leukemia.

In this review article, different types of nanoparticles, their characteristics, and their efficiency in targeting leukemic cells are surveyed, and with the aim of delineating the future of nanomedicine-based therapy. At the end, we discuss clinically approved nanoparticles as the new means of treatment in leukemia.

## 2. Nanoparticles as Drug Delivery Systems

The concept of nanotechnology was first introduced by the physicist Robert Feynman in 1959 during a talk he delivered at Caltech University [10]. Since then, nanotechnology enormously grew in a number of fields, including physics, biology, chemistry, medicine, electronics, and information technology, and it is currently part of our everyday life. The advent of nanotechnology in the medical field, giving rise to the term nanomedicine, dates back to the 1990s [11], even if several nanosystems, now applied for drug delivery, were designed and developed previously in the 1960s [12]. The term ‘nanosystem’ refers to a system having a size comprised between 1 and 1000 nm [11]. They are mostly designed for drug delivery (triggered or non-triggered), imaging, regenerative, and gene therapy purposes. The great advantages of nanosystems are their ability to deliver a high amount of an agent (drug, chemical, or biological product) at the desired site, generally increasing its stability and its blood circulation lifetime while decreasing its side effects (for instance to systemic effects associated to chemotherapeutic drugs) [13,14]. Moreover, according to the composition of the system, nanotechnologies allow to encapsulate and deliver hydrophobic molecules, generally difficult to be freely administered, increasing their solubility and biocompatibility [15,16]. Last but not least, the possibilities of surface modification of nanosystems are enormous, paving the way to the targeting of selected receptors. This aspect is of paramount importance in the era of personalized medicine and will be more thoroughly explored in the next paragraph.

To be eligible for in vivo applications, nanosystems must be composed of materials that are biocompatible, non-toxic, and biodegradable [17]. Moreover, they should be stable after administration and, in view of possible clinical translation, their preparation as easily up-scaled for manufacturing with a high control over their physicochemical properties [14]. Most of the nano-therapeutic products currently approved by the FDA are meant for parenteral administration; only a few preparations have been designed for topic or oral administration. The first nanosystem approved was AmBisome^®^ (Gilead Sciences, Inc., Foster City, California, US), a liposomal formulation of amphotericin B for the treatment of fungal infections [18]. The advantage of amphotericin B encapsulation into high transition temperature phospholipids and cholesterol improved drug’s efficiency and reduced its systemic toxicity [19]. Nowadays, the majority of nanosystems on the market address cancer treatment (Table 1), but other applications include mental illness, anemia, and inflammatory diseases such as rheumatoid arthritis, inflammatory bowel disease, asthma, multiple sclerosis, and diabetes [20]. In the following subsections, an overview of the main nanotherapeutic systems currently available will be given.

### 2.1. Liposomes

**Figure 1:**
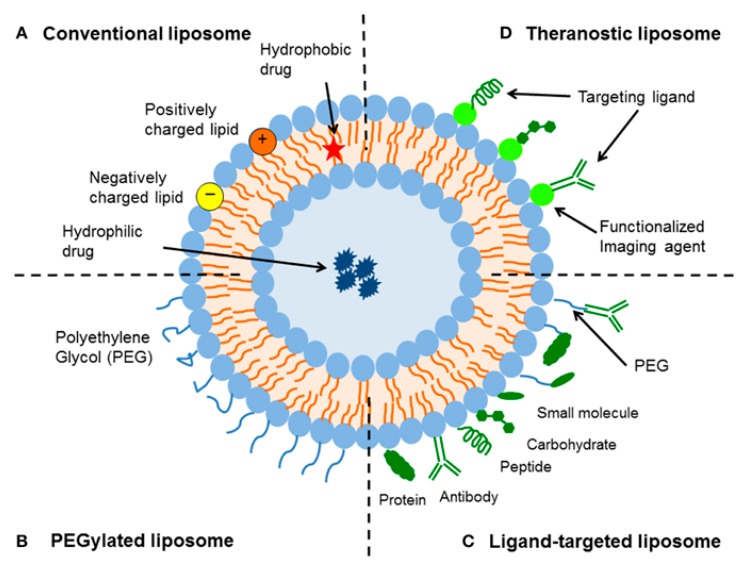
Schematic representation of the different types of liposomal drug delivery systems. (A) Conventional liposome: liposome composed of a lipid bilayer of anionic, cationic or neutral phospholipids and cholesterol. Drugs can be incorporated both in the bilayer (hydrophobic drugs) and in the aqueous core (hydrophilic drugs). (**B**) PEGylated liposomes: lipid bilayer endowed with a PEGylated phospholipid to make the nanosystem stealth and sterically stable. (C) Ligand-targeted liposome—Liposomes can be used for specific targeting by attaching ligands (e.g., antibodies, peptides, and carbohydrates) to its surface or to the terminal end of the attached PEG chains. (D) Theranostic liposome—A lipid bilayer bearing at the same time an imaging and a therapeutic agent. A targeting vector can also be introduced. Reproduced with permission from [23], licensed under CC BY.

Liposomes were first discovered by Bangham et al. [12]. Since then, the research in the field of these nanosystems developed enormously, and they are now considered to be the most successful drug-carrier system known to date [21]. Liposomes are bilayered vesicles, composed of an aqueous core surrounded by one (small unilamellar vesicles, SUVs; or large unilamellar vesicles, LUVs) or more (multilamellar vesicles, MLVs) bilayers of phospholipids [22]. Phospholipids are amphiphilic molecules composed of a hydrophilic head and one or two hydrophobic tails. These molecules spontaneously tend to re-arrange in an aqueous environment to form a structure similar to that of biological membranes [21]. Liposomes are extremely versatile nanocarriers (Figure 1) as they can load both hydrophilic and hydrophobic molecules. Hydrophilic molecules are generally encapsulated into the aqueous core, while hydrophobic ones are usually entrapped into the bilayer, among the hydrophobic tails [16]. When the drug is loaded into these systems, it is not bioavailable. Therefore, the strong advantages of drug encapsulation into liposomes resides in (i) its protection against naturally occurring phenomena, such as enzymatic degradation and immunologic and chemical inactivation; (ii) prevention of its metabolization before reaching target tissues; (iii) reduced exposure of healthy tissue to the drug; and (iv) increased blood circulation life time [23,24,25]. Taken together, all these effects generally contribute to achieving an increased therapeutic index [26].

The composition of phospholipids can vary considerably, affecting liposome properties. The type of phospholipids used, for example, can influence the liposomal surface charge. Neutral liposomes are generally less stable and tend to aggregate; moreover, they do not interact much with cells, thus releasing their cargo in the extracellular environment [27]. Among commonly used neutral phospholipids, we can cite dioleoyl phosphatidylethanolamine (DOPE), dioleoyl phosphatidylcholine (DOPC), or dipalmitoyl phosphatidylcholine (DPPC). Anionic liposomes are generally composed of negatively charged lipids, such as dimyristoyl phosphatidylglycerol (DMPG) and dipalmitoyl phosphatidylglycerol (DPPG). The development of negatively charged liposomes for parenteral administration, however, met with some drawbacks basically associated to their interaction with biological systems, with consequent drug release, toxic effects, and fast opsonization by complement and other circulating proteins, followed by liposome uptake by the immune system cells [28]. Cationic liposomes, instead, were first described by Felgner et al. in 1978 [29], and are currently designed mainly for gene delivery purposes, due to the occurrence of stable electrostatic interaction between negatively charged nucleic acids and positively charged phospholipids [30]. Among phospholipids employed to obtain cationic liposomes there are 1, 2-dioleoyl-3-trimethylammonium-propane (DOTAP), dioleoylphosphatidyl ethanolamine (DOPE), dimethyldioctadecylammonium bromide, N-[1-(2,3-dioleoyloxy) propyl]-N,N,N-trimethyl-ammonium methyl sulfate and oleic acid (OA). The addition of cholesterol into the phospholipid bilayer can decrease the permeability of the liposome membrane and increase its *in vitro/in vivo* stability, thanks to the dense phospholipid-packing effect exerted by this hydrophobic molecule [31]. Another component often employed for liposome formulation are pegylated phospholipids: lipids modified with polyethylene glycol (PEG). PEG is a non-toxic and non-ionic hydrophilic polymer that confers to liposomes higher stability and extended blood circulation time, due to the reduced uptake by immune system cells [32]. It acts as a steric barrier, hindering the interactions between the nanosystem and serum protein that are involved in recognition of the carriers by the mononuclear phagocyte system. This steric stabilization was reported to increase blood half-life of liposomes from 2 h up to 24 h in rodents (mice and rats) and as high as 45 h in humans, depending on the particle size and the characteristics of the coating polymer [23]. Finally, specific phospholipids or molecules can be included in the liposomal formulation to achieve triggered release under certain conditions (e.g., temperature, pH, enzymes, light, ultrasounds). Temperature triggered drug release is based on the phase transition temperature (T_m_) of phospholipids. T_m_ is defined as the temperature at which a transition occurs from an ordered gel phase to a disordered fluid phase [33]; during this transition, the liposomal payload is generally released, due to the loosening of the tightly packaging of the phospholipid bilayer. The first thermosensitive liposomes developed were mainly composed of phosphatidylcholines, bearing a T_m_ in the range of mild hyperthermia (40–43 °C) [34]. While, ThermoDox^TM^, a liposomal formulation containing doxorubicin currently in a clinical trial for the treatment of hepatocellular carcinoma (clinicaltrials.org identifier: NCT00617981), exploits the lysolipid thermally sensitive liposome technology to encapsulate doxorubicin and release it selectively at 41.3 °C, thanks to pore formation into the membrane [35,36]. Thus, the release can be easily localized only in artificially heated regions (e.g., tumor region).

### 2.2. Micelles

**Figure 2:**
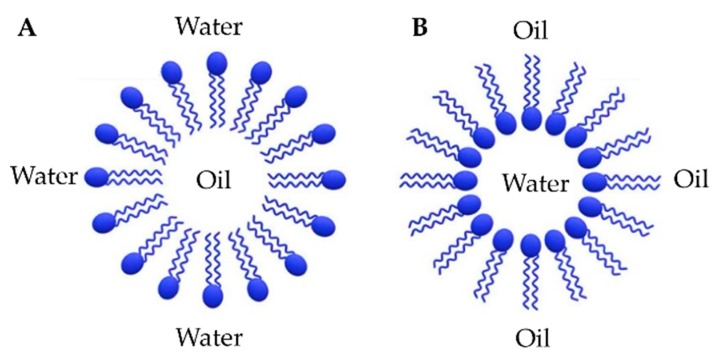
Schematic representation of (**A**) normal micelles and (**B**) inverted micelles. Adapted from [38] (published by MDPI), licensed under CC BY.

Micelles are another type of biocompatible nanosystems, with a size comprised between 5 and 100 nm. They are composed of a monolayer of amphiphilic molecules that spontaneously tend to self-assemble in aqueous environments at a definite concentration, known as critical micelle concentration (CMC). These amphiphilic molecules are generally fatty acids, salts of fatty acid (soaps), phospholipids, or other similar amphiphilic compounds [37]. Micelles present either a hydrophobic core, exposing outside the hydrophilic polar heads, or a hydrophilic core, exposing outside the hydrophobic tails (inverted micelles) (Figure 2) [38]. They usually encapsulate hydrophobic drugs into the hydrophobic core, whereas hydrophilic drugs can be adsorbed or chemically linked to the outer shell [39]. The first method of encapsulation is generally less stable, as these structures can rapidly de-assemble after intravenous injection, due to both a dilution effect and interactions with surfactant proteins. To overcome this drawback, many strategies have been proposed, among which are the inclusion of a crystalline copolymer and a copolymer with a lower critical micellar concentration in the formulation, or the crosslinking of the core and/or shell regions [40]. The delivery of anti-cancer drugs within biocompatible micelles in comparison to free drug administration resulted in reduced systemic toxicity and increased drug solubility as well as site-specific tumor accumulation [41].

### 2.3. Polymeric Nanoparticles

Polymeric nanoparticles are either solid nanospheres or nanocapsules displaying a size of 1–1000 nm. They can be composed of either synthetic polymers, such as poly(lactide), poly(lactide-co-glycolide), and poly(ε-caprolactone), or natural polymers like chitosan, alginate, gelatin, and albumin [42]. These polymers must be biocompatible and biodegradable. Drugs can either be dispersed within the polymer matrix or directly conjugated to the polymer molecule. Drug release can occur in different ways: diffusion, swelling of the polymer matrix, or polymer erosion, and degradation [43]. In general, synthetic polymers allow for sustained drug release, within a period of days to weeks, while natural polymers are more easily and rapidly degraded. The most diffuse biodegradable polymer used for the preparation of nanoparticles is poly(lactic-co-glycolic acid) (PLGA) [44]. PLGA is an FDA approved copolymer of poly lactic acid (PLA) and poly glycolic acid (PGA), displaying a wide range of erosion times and tunable mechanical properties [45]. According to the molar ratio of PLA and PGA used for polymerization, different forms of PLGA can be obtained. PLGA Nanoparticles have been widely used in preclinical investigations for the encapsulation and delivery of various anticancer drugs, however, so far, no anticancer PLGA formulations have been approved by FDA.

Poly(ε-caprolactone) is another FDA approved polymer used to prepare nanoparticles. It is obtained from the monomer ε-caprolactone and, in comparison to PLGA, display a slower degradation time both *in vitro* and *in vivo*, thus being more suitable for long-term delivery systems. Many poly(ε-caprolactone) Nanoparticles have been synthesized for cancer therapy displaying very high encapsulation efficiency values [46,47].

Chitosan is a natural biodegradable, biocompatible polymer obtained by the partial *N*-deacetylation of chitin. It can be easily functionalized, resulting in a broad portfolio of possible applications. It has reduced toxicity and has been often used for the preparation of nanoparticles containing anticancer drugs [48,49]. Nanoparticles prepared with chitosan and chitosan derivatives, in fact, typically possess a positive surface charge and mucoadhesive properties that facilitate their adhesion to mucous membranes and the release of the drug payload in a sustained release manner [50]. Moreover, due to their positive charge, they can be used as non-viral vectors for gene therapy. Chitosan based polymeric nanoparticles are generally designed for non parenteral administration.

Another type of polymeric nanoparticles are nanogels, also known as hydrogels nanoparticles, with a size generally comprised between 20 and 200 nm and a large surface area [50]. In the last decade, much research has been devoted to the development of these nanosystems. Nanogels can be obtained by physical or chemical cross-linking of synthetic hydrophilic polymers or biopolymers. Physical cross-linking, being non-covalent, is considered to be less stable, thus requiring additional stability assays before *in vitro/in vivo* experiments. The porous network resulting from the cross-linking allows for high drug entrapment efficiency. The advantages of using nanogels in comparison to the free drug are enhanced drug stability, prolonged blood circulation time, and the possibility to respond to specific stimuli to obtain drug release (pH, magnetic field, light, ionic content, and temperature) [51]. Moreover, their high water content and living tissue-like physical properties ensure high biocompatibility. Nanogels meant as anticancer drug delivery systems have been recently designed [52,53].

### 2.4. Solid Lipid Nanoparticles (SLNs)

Solid lipid nanoparticles (SLNs) are systems with a size of 50–1000 nm, composed of lipids that are solid at body temperature (fatty acids, steroids, waxes, monoglycerides, diglycerides or triglycerides), surfactants, as stabilizers, and sometimes co-surfactants. SLNs combine the advantages of emulsions and liposomes with those of polymeric nanoparticles (like protection of incorporated drugs from degradation, controlled release, excellent tolerability), while simultaneously avoiding some of their disadvantages such as in vivo stability problems [54]. Additionally, SLNs can overcome several physiological barriers that hinder drug delivery to tumors and are also able to escape multidrug resistance mechanisms, characteristic of cancer cells. Hydrophobic drugs can be easily trapped in the lipidic matrix, while hydrophilic drugs must be chemically conjugated to the lipidic components or to pegylated phospholipids that are sometimes added in order to obtain steric hindrance and enhance SLNs blood circulation time [55]. Potential disadvantages of SLNs are their insufficient drug loading capacity (around 25–50% in comparison to the lipid matrix) and the possible accidental drug expulsion during long-term storage [56]. These drawbacks are generally caused by the formation of the crystal solid state of the lipids into the matrix, depending on the selection and relative proportion of the components, as well as on the preparation method. Due to possible post-production polymorphic transition of the crystals, in fact, drug expulsion and particle instability can occur. To increase drug-loading capacity, lipids forming non-ordered crystals are then preferred (mono-, di-, triglycerides, with different chain lengths). Moreover, to facilitate drug encapsulation and boost the in vivo drug release rate, the addition of one or more lipids liquid at room temperature (like oleic acid, for example) to SLNs formulation can be envisaged, obtaining the so-called nanostructured lipid carriers (NLCs) [57]. Depending on the amount of liquid lipid, its insertion will give rise to amorphous or partially crystalline solid matrices. Another critical aspect in the formulation of SLNs and NLCs is the choice of surfactant molecules, which should be compatible with the employed lipids. Among the amphiphilic molecules used as surfactants, we can cite monoacylglycerides of long-chain fatty acids, phospholipids, some esters, poloxamers, and polisorbates. Co-surfactant, like bile salts such as taurodeoxycholate, or alcohols such as butanol or ethanol, can be added to further increase the stability of SLNs. The use of SLNs as vehicle for anticancer drugs has been widely explored and resulted in greater cytotoxic capacity than that of free drugs, enhanced drug bioavailability, and reduced systemic side effects [58,59]. Finally, it is worth mentioning that, in view of eventual clinical translation, large-scale production of SLNs is technically and economically feasible.

### 2.5. Inorganic Nanoparticles

**Figure 3:**
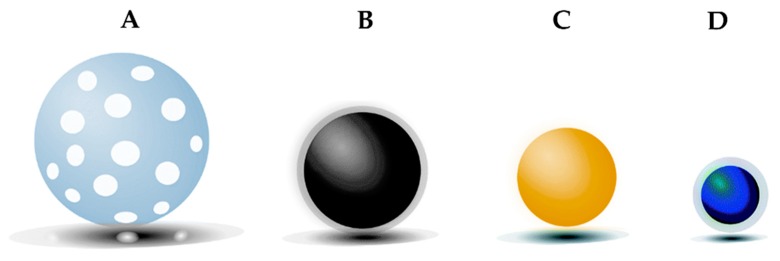
Main types of inorganic nanoparticles: (**A**) silica nanoparticles, (**B**) iron oxide nanoparticles, (**C**) gold nanoparticles, (**D**) quantum DOTs. Adapted from [63].

Inorganic nanoparticles are systems composed mainly of inorganic compounds and pure metals. The most investigated ones are quantum dots, gold, iron oxide, and silica nanoparticles (Figure 3). Gold nanoparticles are characterized by low toxicity, peculiar optical and electrical properties, potential biodegradability, and considerable surface modifiability, thus representing a highly feasible material for treating malignant tumors. They have a size of 1–150 nm and are generally composed of a gold atom core that can be functionalized by the addition of a monolayer of various moieties containing a thiol group [60]. A PEG coating can also be added to these nanoparticles to make them stealth and increase their blood half-life. They are used in preclinical investigations and clinical trials as drug delivery carriers [61], in photodynamic therapy for the treatment of cancer or imaging purposes [62].

Iron oxide nanoparticles can display ferromagnetic or superparamagnetic (preferred in the medical field) properties. Their size ranges from a few nm to microns. They have been widely investigated mainly for magnetic resonance imaging (MRI) purposes and the treatment of anemia, obtaining the approval of the FDA. Moreover, they can be exploited to induce local heat enhancement (hyperthermia) when submitted to an alternative magnetic field in order to selectively kill cancer cells [64]. Iron oxide nanoparticles are considered biocompatible, stable, and biodegradable systems. Their usage for drug delivery purposes has been investigated only in recent years. In general, they are composed of an inorganic core made of magnetite (Fe_3_O_4_) or maghemite (γ-Fe_2_O_3_) surrounded by an (in)organic coating that must ensure stability and stealth in biological media. Moreover, their surface can be modified in order to make the nanoparticles responsive to a specific stimulus (change in pH, temperature, or redox state) and trigger drug release [65]. The great advantage of iron oxide nanoparticles, in comparison to other types of nanocarriers, is the possibility to guide drug release magnetically.

Silica nanoparticles are highly biocompatible, biodegradable, and chemically stable nanosystems (size of 10–10,000 nm), displaying an easily tunable mesoporous structure (2–50 nm pore size). Their solid framework, composed of Si-O bonds, is extremely resistant to degradation or external stresses. Thanks to their high surface to volume ratio, they display elevated drug loading capacity as well homogeneous drug loading. Moreover, various functional groups can be easily attached to the particles in order to target them to a particular site or to make them responsive to a specific stimulus, in order to trigger drug release [66]. The major drawback of these systems resides in their hemolytic effect, due to the favorable interaction between their silanol groups and the surface of the phospholipids of red blood cell membranes [67]. Since their first introduction by Vallet-Regi and co-workers in the early 2000s, many silica nanoparticles have been designed for cancer drug delivery purposes [68].

Finally, even if used mainly for imaging purposes, also quantum dots (QDs) should be mentioned among inorganic nanoparticles used for cancer therapy. QDs are small nanoparticles (2–50 nm in diameter) consisting of semiconducting material (like Cd, Se, Zn, Te, Hg, and Pb), displaying unique optical and electronic properties [39]. The primary issue for their possible clinical translation is their toxicity, associated with the presence of heavy metals and their colloidal instability [69].

## 3. Targeted Cancer Therapy: Advantages in Comparison to Conventional Treatments

The idea of targeted therapy was introduced by Paul Erlich in the first 1900s, with the so-called “magic bullet concept”: drugs should go straight to their intended cell targets, thus efficiently killing pathogens, while being harmless in healthy tissues [70]. This concept initially referred mainly to infectious diseases was then extended to cancer treatment. The first breakthrough in cancer targeted therapy dates back to World War II when the injection of mustin, the prototype of nitrogen mustards (cytotoxic anti-cancer agents), to a patient with non-Hodgkin lymphoma caused a robust anti-tumor effect [71]. Erlich thus assumed that small molecules could be useful in the treatment of cancer. Later on, in 1948, S. Farber, now known as the “father of modern chemotherapy”, observed that folic acid yielded a proliferative effect on cancer cells when administered to children suffering from acute lymphoblastic leukemia (ALL). According to this observation, he synthesized antagonists of folic acid that proved to be effective in the treatment of this pathology [72] and became the mainstays of leukemia chemotherapy. In the 1970s–1980s, the second wave of cancer-targeted therapeutic drugs was proposed. During these years, various small target molecules were designed in order to hit specific mutations identified as driving forces of cancer progression. Among plenty of agents developed to target crucial effectors involved in cell proliferation, invasion, metastasis, angiogenesis, and apoptosis [73], imatinib was synthesized [74]. Imatinib, a tyrosine kinase inhibitor, now considered as a milestone drug, proved to be effective in the treatment of chronic myeloid leukemia (CML) [75]. One step further was made with the development of Sunitinib, a multi-targeted chemotherapeutic. This drug was developed, starting from the consideration of cancer as a multi-factorial disease. Sunitinib targets simultaneously all receptors for platelet-derived growth factors (PDGF-Rs) and vascular endothelial growth factor receptors (VEGFRs), which play a role in both tumor angiogenesis and tumor cell proliferation, and also CD117, a tyrosine kinase receptor that (when improperly activated by mutation) drives the majority of gastrointestinal stromal cell tumors receptors namely CD114, CD135, and RET [76]. Finally, the latest breakthrough has been applied for the production of monoclonal antibodies, as shown by Georges Köhler and César Milstein in 1975 [77,78]. Erlich already predicted that “antibodies are in a way magic bullets that identify their target themselves without harming the organism” [73]. However, the first murine antibodies administered to humans presented, many drawbacks mainly linked to immune reactions and reduced ability to induce immune effector mechanisms [79]. In the years that followed, a number of chimeric and humanized antibodies able to attack cancer cells with various strategies, such as antibody-dependent cellular toxicity, complement-dependent cytotoxicity, modulation of signal transduction, and immunomodulation, were developed [80,81]. The first successful monoclonal antibody approved by the FDA (in 1998) for the treatment of breast cancer was trastuzumab, an antibody able to specifically target ERBB2 (also known as HER2) receptor, overexpressed in breast and ovarian cancer [82]. In the same years, Rituximab was also approved by the FDA. Rituximab is a genetically engineered chimeric mouse-human antibody that binds to the transmembrane antigen CD20, involved in B-cell non-Hodgkin lymphoma. Since its initial approval in 1997, it has improved outcomes in all B-cell malignancies [83]. Nowadays, many drug-antibody conjugates are under development, in order to merge the advantages of monoclonal antibodies with chemotherapeutic drugs, while at the same time eluding drug resistance mechanisms. Taken together, all these discoveries have contributed to substantial progress in cancer therapy. One step further has been made by conjugating these targeting strategies to the use of nanosystems, already in the clinical practice since the 1990s. As illustrated in the previous paragraph, nanosystems have the advantage of protecting the encapsulated drug from degradation, bringing a high payload of a drug at the site of interest, increasing its blood circulation time, and delivering it more specifically to cancer cells, through passive or active targeting.

### 3.1. Passive Targeting

The term ‘passive targeting’ refers to the accumulation of a drug or a drug-carrier system into a specific organ or pathological tissues via biological mechanisms, such as RES (reticuloendothelial system) or EPR (enhanced permeability and retention) effects [84]. The reticuloendothelial system, also known as the mononuclear phagocyte system, consists of cells descending from the monocytes, which can perform phagocytosis of foreign materials and particles. It is part of the body’s defense mechanisms. If the nanoparticles are not appropriately modified, they can be rapidly taken up and retained into the major reticuloendothelial organs (liver and spleen, mainly). At present, the most promising strategies to limit RES uptake consist of reducing particle size (particles with a diameter below 35 nm seems to be less prone to RES uptake) and in sterically stabilizing the nanocarriers utilizing PEG-lipids [85].

**Figure 4:**
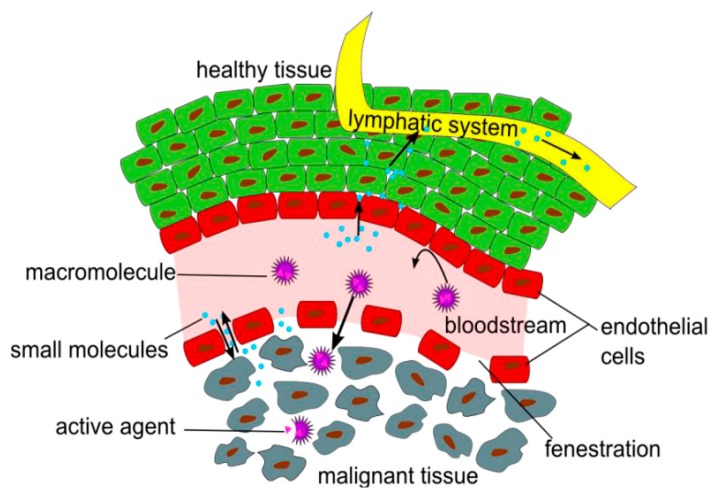
Schematic representation of the EPR effect. Reproduced with permission from [87] (published by MDPI), licensed under CC BY 3.0.

The EPR effect, first described in 1986 by Matsumura and Maeda, is based on anatomical differences between healthy and tumor vasculature. The fast and disordered tumor growing process lead to the development of vasculature with a quite abnormal architecture, characterized by leaky blood vessels, with gaps as large as 600–800 nm, and poor lymphatic drainage [86]. This allows the extravasation of macromolecules through these gaps into the extravascular space and their accumulation inside solid tumor tissues (Figure 4) [87]. The EPR effect results in a substantial increase in tumor drug storage in comparison to healthy organs and in dramatically enhanced, usually 10-fold or higher, tumor drug accumulation, when it is delivered by a nanoparticle rather than as a free drug. [88]. It should be noted that the EPR effect can vary considerably as it is affected by various factors, such as tumor size, tumor perfusion, variation in endothelial gap size (ranging from one to hundreds of nanometers) and nanoparticle characteristics (e.g., size, surface charge, shape, etc.). Moreover, it should be noted that passive targeting cannot be used as a strategy to deliver Nanoparticles to treat hematological malignancies (i.e., leukemia and lymphoma) and metastatic lesions, as under these circumstances EPR effect does not play any role [89]. In hematologic malignancies, in fact, tumor cells are free in circulation, thus requiring specific active targeting. However, confined tumor sites, such as the bone marrow and/or lymphoid tissues, in which the EPR effect can be exploited are also present [90].

### 3.2. Active Targeting

The term ‘active targeting’ refers to the conjugation of targeting moieties on the surface of nanocarriers for the crossing of biological barriers, specific tumor homing, increased retention at the target site, and uptake by target cells. When designing actively targeted nanoparticles some factors must be considered in order to maximize the targeting efficiency. First, the targeted antigen or receptor should be expressed solely on tumor cells and not on healthy cells to minimize or avoids off-target effects of the active therapeutic agents on healthy tissues. Second, they should be expressed homogeneously on the targeted tumor cells. Third, the recognition between the receptor and the targeting moiety should not be prevented in blood circulation, for example, due to protein corona adsorption onto the nanocarrier [91]. Finally, to improve treatment efficiency, it is vital that not only the receptor-targeting ligand recognition takes place but also that the internalization of the nanocarriers occurs [88]. Targeting moieties include peptides, protein domains, antibodies, aptamers, and small molecules, such as folate or growth factors.

The major advantages of using small natural molecules as targeting ligands reside in their stability, ease, and density of conjugation at the surface of nanoparticles; potential low cost; lower molecular weight; and lower immunogenicity than antibodies. However, some of these ligands, such as folate, transferrin, or carbohydrates (like lactose, galactose, galactosamine, mannose, glucose, trehalose, and hyaluronic acid), naturally display quite high concentration in human body as they are supplied by food intake, thus competing with the targeted nanocarrier in receptor binding and consequently reducing specific drug delivery [92,93].

Polypeptide-based moieties—including peptides, protein domains, and antibodies—are among the most diffuse targeting ligands. Antibodies are highly specific ligands; they can be used as both therapeutic and targeting agents [94]. Their big size, while favoring the specific interaction with the targeted receptors, limits their density on the nanoparticle surface as well as can induce immunogenicity and considerable RES uptake. In the last decades, many nanoparticles functionalized with antibody have been designed, among which are immunoliposomes, composed of liposomes entrapping one or more anticancer drugs, functionalized with monoclonal antibodies, like anti-HER2 or anti-EGFR antibodies [95,96]. Smaller antibodies, known as affibodies, can also be used as targeting ligands. Affibodies are engineered, robust, high-affinity proteins designed to bind a large number of target proteins or peptides, mimicking monoclonal antibodies. In contrast to antibodies that have the Fc-portion, however, they lack half-life extension and effector function modules [97].

Peptides are defined as linear or cyclic amino acid sequences with less than 50 residues [98]. In comparison to proteins, peptides display a series of advantages, such as the ease and higher density of conjugation to nanocarrier surface, higher stability, reduced immune reaction, lower cost of manufacturing, and enhanced feasibility of large-scale production. However, the binding specificity is limited in comparison to that of antibodies [99]. The most studied tripeptide motif is arginylglycylaspartic acid (RGD). It can specifically bind αvβ3 and αvβ5 integrin receptors, which are overexpressed on both tumor endothelial cells and cancer cells [100].

Aptamers, instead, are small single-stranded nucleic acids that fold into a well-defined three-dimensional structure. They can bind to a variety of targets with high affinity and specificity and inhibit their biological functions. The advantages of aptamers as targeting moieties in comparison to antibodies are their reduced size, high in vivo stability, non-immunogenicity, and limited toxicity [101]. At the same time, however, their successful employment in clinics is still challenging because of their short blood half-life, due to degradation by serum nucleases and rapid renal clearance. Moreover, the aptamer-receptor binding is highly dependent on their three-dimensional structure, which can be influenced by surrounding environmental conditions [102].

Even if the portfolio of available targeting ligands is extremely wide and can be specifically tailored to each disease, the real power of targeting nanomedicine is still under debate. A recent meta-analysis of pre-clinical data on nanoparticle-based delivery platforms for malignant tumors published over the past ten years reported that only an average of 0.9% of the injected dose of active targeted nanocarriers reaches the target tumors [103]. This value seems to be extremely low, however, it is already two or three times more than what is obtained with the free drug and almost 1.5-times more than passive-targeted systems (0.9% vs. 0.6%). In relative terms, this represents a substantial increase in delivery efficiency and establishes the advantage of nanoparticles for tumor-targeted drug delivery. However, in order to further increase this percentage values, some approaches can be explored. As tumors are generally extremely heterogeneous targeting only a single cell-surface receptor can result in partial tumor response, which in most cases is followed by a resistant tumor relapse and mortality. The development of a novel class of nanoparticles with versatile targeting moieties or bearing different targeted ligands simultaneously has been proposed [89].

## 4. Nanosystems in the Treatment of Myeloid Malignancies

Acute myeloid leukemia (AML), the most common form of leukemia in adults, is characterized by abnormal proliferation, block of differentiation, and infiltration of immature cells into blood and bone marrow [104]. Treatment of AML, consisting of continuous infusion of cytarabine and anthracycline in the 7 + 3 regimen, has not been changed from past decades [105]. In this case, we are facing an overall survival (OS) of about 40% in younger patients and inferior results for patients who are older than 60 years of age [106]. The addition of new FDA approved compounds such as Midostaurin, and Giltritinib for targeting Fms-related tyrosine kinase 3 (FLT3) mutated AML, Enasidenib, and Ivosidenib for isocitrate dehydrogenase 1 and 2 mutated AML, and also Venetoclax as BCL2 inhibitor, has been carried out to ameliorate the outcome [107,108].

Patients with therapy-related AML (t-AML) as the result of previous chemotherapy or radiation [109] and patients with the history of myelodysplastic related changes (AML-MRC) [110] account for 10–20% of the AML cases. Albeit these patients have been removed from many clinical trials due to their inferior prognosis, a nano-based approach called CPX-351 was developed in 2017 for the treatment of these subgroups [33]. CPX-351 is a liposomal formulation of combinatorial therapy composed of cytarabine and daunorubicin in a fixed 5:1 molar ratio. The liposome membrane is comprised of distearoylphosphatidylcholine, distearoylphosphatidylglycerol, and cholesterol in a 7:2:1 molar ratio. Encapsulation of the above drugs into the liposome has several advantages over conventional therapy, such as increment of blood circulation half-life, reduction of toxicity, and above all, maintaining the molar ratio, among the administered drugs which guarantees the synergistic effect and may circumvent antagonistic effect [111,112]. In a randomized clinical trial phase 2, the efficacy of CPX-351 and conventional 7 + 3 regimens in newly diagnosed t-AML and AML-MRC was compared. While the target was OS, using CPX-351 notably increased median OS compared to 7 + 3 regimens (9.56 months compared with 5.95 months). Meanwhile, attaining a higher complete remission in patients who received CPX-351 demonstrates the power of this approach [113].

FLT-3, which accounts for 30% of AML cases, is associated with poor prognosis. This tyrosine kinase receptor promotes survival, proliferation, and differentiation blockade in leukemic cells by activating downstream signaling pathways [114,115]. Jiang et al. used polyamidoamine dendrimers conjugated with FLT-3 ligand loaded with microRNA(miR)-150 as a tumor suppressor. This system, by selectively targeting FLT-3 mutated cells, exhibited a profound anti-tumor effect both *in vitro* and in vivo and negated off-target interactions [116]. It has been shown that miR-29b is downregulated in AML and is considered as a tumor suppressor microRNA [117]. In one study, targeted delivery of miR-29b via lipopolyplex nanoparticle conjugated transferrin led to the downregulation of some genes participating in leukemogenesis and brought about a higher survival rate in the mouse model of AML [118]. Some studies demonstrated that nucleolin, a multifunctional protein involved in metabolism and gene regulation, has a higher expression on the surface of the leukemic cells [119]. Deng et al., in order to exploit this feature, designed a gold nanoparticle loaded with doxorubicin conjugated with an anti-nucleolin aptamer called AS1411 and anti-miR-221 as an oncogenic miR. This multifunctional nanoparticle revealed a high efficiency in targeting resistant leukemic cells [120].

In another subtype of AML, acute promyelocytic leukemia (APL), the preferred type of treatment is germane to using all-trans-retinoic acid (ATRA) in combination with arsenic trioxide or chemotherapy agents [121]. In recent years, a novel synthetic retinoid called ST1926 has been produced to target APL and also other forms of leukemia [122,123]. ST1926 displayed lower toxicity and is regarded as a more potent agent in inducing apoptosis and controlling cell growth. However, due to lower bioavailability and fast excretion, El-Houjeiri et al., loaded ST1926 in polystyrene-b-poly ethylene diblock copolymer. The drug-loaded NP appeared to have enormous potential in reducing leukemic cell proliferation (*in vitro*) and diminishing tumor burden and enhancement of survival in a mouse model of AML [124].

CD33 is commonly expressed in leukemic cells compared to healthy hematopoietic stem cells it represents a good alternative for targeted therapy. Many experiments using anti-CD33 antibody and conjugation with liposome or lanthanide oxyfluoride nanoparticle reported an effective way to target CD33 expressing leukemic cells [125,126].

Although most of the conventional drugs and designed nanoparticles are dependent on the elimination of the bulk disease population, a small and rare population called leukemic stem cells (LSC) may be able to resist the therapy and may relapse after a while [127,128]. These leukemic stem cells, after facing ineffective therapies, evolve and change their characteristics and make a new population of LSC [129]. In such circumstances, eradication of LSC has to be tackled with alternative approaches. The population of LSC in AML is heterogeneous, and the expression of CD markers such as CD44, CD47, CD93, CD96, CD123, CLL-1, TIM-3, etc., is variable from patient to patient. Moreover, some of these markers, such as CD44 and CD47, are also expressed on normal stem/progenitor cells, thus finding specific markers of AML LSC using various techniques such as flow cytometry and cell surface capturing technology for targeted therapy by nanoparticle seems practical [127,130].

In the study devoted to target AML LSC, Lin et al. produced nano-micelles loaded with daunorubicin and conjugated it with a CLL-1 targeting peptide. They reported higher toxicity on leukemic stem cells and lower serial transplantation capability in mice as the representation of the self-renewal feature of leukemic stem cells [131]. Parthenolide (PTL) also exerted an inhibitory effect on leukemic stem/progenitor cells through the upregulation of P53 as a tumor suppressor and downregulation of NF-kβ as a pro-survival transcription factor. However, due to low bioavailability and poor solubility, micelle and nanoparticle loaded PTL appeared good systems to eliminate leukemic stem cells selectively [132,133].

In most cases, the nanomedicine in leukemia has been used to solve the problem of lower bioavailability and higher toxicity, but it can be used in several ways to improve the treatment in AML. Namely, i) using nanocarriers loaded with different agents for combination therapy to increase drugs half-life and to keep the different drugs molar ratios fixed; ii) targeting selectively a resistant subpopulation, which may differ from patient to patient; iii) using nanosystems concomitantly with conventional therapy to eradicate bulk disease population and leukemic stem cells simultaneously.

CML is a hematopoietic stem cell malignancy that results from a translocation between the Abelson gene (ABL1) on chromosome 9 and break point cluster region (BCR) gene on chromosome 22. The encoded protein governs the disease by activating downstream signaling pathways. However, absent effective therapy second mutation transforms the chronic phase to the acute phase, which is associated with a higher proliferation rate and impairment of differentiation [134,135]. Treatment of CML is based on applying different generation of tyrosine kinase inhibitors (TKIs) such as imatinib, nilotinib, and dasatinib. In half of the CML patients who achieve a deep molecular response, therapy can be halted without leukemic recurrence, a term called treatment-free remission (TFR). However, it appears possible to increase the number of patients who achieve TFR, including the case of resistance due to domain mutations and the presence of CML LSCs, which are insensitive to TKIs treatment [134,136]. In this context, Liu et al. encapsulated homoharringtonine, a drug for patients resistant to TKIs, into PEGylated liposome, and they demonstrated comparable safety and lower toxicity as opposed to the bare drug [137]. In another study, loading bortezomib into liposome conjugated to transferrin significantly targeted CML resistant cells to doxorubicin and made them more sensitive to this agent [138]. Jyotsana et al. designed a liposome loaded with siRNA against BCR-ABL1 and claimed that this system could decrease the disease burden in the mouse model of CML [139]. As many markers and molecular participants in the pathogenesis of CML have been discovered, it is expected they may open up an opportunity to develop a nanomedicine-based therapy to optimize the treatment strategy.

## 5. Nanosystems in the Treatment of Lymphoid Malignancies

ALL is the most common form of cancer in children with an incidence rate of 1.6 per 100,000 persons [140]. ALL follows a bimodal distribution with the main peak in children and the second peak in patients who are older than 50 years of age [141]. While some genetic abnormalities such as down syndrome, Fanconi anemia, or even environmental factors augment the risk of ALL, in most cases, it is regarded as a de novo disease [142]. The treatment strategy is based on employing chemotherapy agents such as vincristine for induction, consolidation, and maintenance. By using conventional therapies, the cure rate in children is satisfactory with a 5-year OS of about 90%, while in older patients, the response rate falls off and is between 30–40% [143]. The occurrence of patients who are resistant to first induction therapy and patients who relapse at any stage of the treatment is the major obstacle of the effective treatment and salvage therapy based on CAR T-cells, and targeted therapy by antibodies against CD19 (such as coltuximab ravtansin), CD20 (such as rituximab), and CD22 (such as inotuzumab ozogamicin) appears to be the best viable option [140]. Vincristine, as a chemotherapy drug, has been employed in different steps of ALL treatment and even as salvage therapy. However, the administration of this agent is accompanied by toxicities such as neuropathy, which increases in a dose-dependent manner [144,145]. To deal with this problem, vincristine sulfate liposome injection (VSLI) was developed. This liposomal formulation increased the biodistribution and half-life of vincristine sulfate and allowed for a higher dose of intensification compared to bare vincristine. In the clinical trial phase 2, patients in the second or greater relapse treated weakly with 2.25 mg/m^2^ of VSLI achieved complete remission of 20% and an overall response rate of 35% [146,147].

Spleen tyrosine kinase (SYK) proceeds through the activation of B cell receptor (BCR) or pre-B cell receptor in B-ALL. It works as a signal transducer and by triggering downstream signaling pathways such as PI3K and NF-kβ acts as a critical element in BCR signaling [148]. Studies reported that, by blocking SYK, cell growth was hampered and apoptosis were induced in the leukemic cells. Uckun et al. demonstrated higher expression of SYK in B-ALL in early relapse and reported that the encapsulation of C61, an inhibitor of SYK phosphorylation, into liposome had a potent anti-leukemic activity [149]. Further study revealed that the combination of this approach with low dose total body irradiation (TBI) could remarkably deplete refractory B-ALL clone and leading to higher OS compared to TBI and C61-liposome alone [150].

CD19 is a transmembrane protein that is expressed by B cell lineage and is a co-receptor of BCR. CD19 in a complex with BCR interacts with downstream tyrosine kinase proteins and has a role in the enhancement of proliferation in leukemic cells. Also, a higher internalization feature after attachment of the antibody makes it a suitable antigen for targeting [151,152]. Another study demonstrated that the conjugation of an antibody against CD19 tagged with C61-liposome displayed higher potency in the eradication of B-ALL and in the reduction of clonogenic activity in vivo due to the lower off-target effect [153]. Conjugating anti-CD19 to norcantharidin loaded liposome also displayed a high specificity and toxicity against CD19 positive cells [154].

Chronic lymphocytic leukemia (CLL) is the most common form of leukemia in western countries with an incidence rate of 4–5 per 100,000 inhabitants. The median age at diagnosis is around 70 years and is recognized by the uncontrolled proliferation of CD5+ B lymphocytes in the blood, bone marrow, lymph node, and spleen [155,156]. As the frontline treatment, chlorambucil, a chemotherapy agent, has been used for several decades, and the development of a combination therapy based on anti-CD20 (rituximab), purine analogue such as fludarabine and cyclophosphamide has improved the treatment [155,157]. It has been verified that higher BCL2 protein in CLL patients increases the resistant to therapy, and like any other form of leukemia, venetoclax as BCL2 inhibitor offered a choice for the treatment [158]. The combination of venetoclax and rituximab in relapsed or refractory CLL patients showed a prominent efficiency, and 2-year progression-free survival rates were 84.9% [159]. G3139 is antisense to the first six codons of bcl2 mRNA. Preclinical and clinical studies revealed that G3139 significantly induces apoptosis and reduces chemosensitivity in many cancers, including CLL [160]. A recent study confirmed that targeting the delivery of G3139 with immunoliposome conjugated rituximab had a therapeutic benefit [161]. In another study, Chiang at al. produced miR-29b loaded immunoliposomes and conjugated this system with an antibody against Tyrosine-protein kinase transmembrane receptor (ROR1). Since ROR1 is mainly expressed by CLL leukemic cells, using this strategy seems a good approach for selective targeting [162]. In summary, studies based on the use of nanomedicine-based therapy in ALL and CLL proved the efficiency of the approach by diminishing side effects and boosting the efficiency of the treatment.

## 6. Clinical Trials

Clinical trials of nanosized systems currently ongoing for the treatment of myeloid and lymphoid malignancies are manifold (Table 2). These trials concern the administration of nanoscaled agents either alone or in combination with other drugs or biological agents. The most diffuse formulation is the CPX-351, also known as Vyxeos^®^ (Jazz Pharmaceuticals, Dublin, Ireland), a liposomal-encapsulated combination of cytarabine and daunorubicin, approved by the Food and Drug Administration (FDA) in 2017 for the treatment of newly diagnosed t-AML or AML-MRC [33]. Cytarabine, also known as cytosine arabinoside (ara-C), is a pyrimidine nucleoside analogue that inhibits the synthesis of DNA, acting on the S phase of the cell cycle. It also has antiviral and immunosuppressant properties. Daunorubicin instead, is an anthracycline topoisomerase inhibitor. Based on the assumption that different drugs administered together can act antagonistically or synergistically depending on the proportional amount of each agent [111], various preclinical studies have been carried out to find the best performing ratio between cytarabine and daunorubicin. Finally, the optimal molar ratio of the two agents encapsulated into the CPX-351 liposomal system was found out to be cytarabine: daunorubicin 5:1 [163]. In the numerous clinical trials in which CPX-351 is currently involved, it is administered either alone or in combination with other drugs/biological agents. An example is the combination of CPX-351 and gemtuzumab ozogamicin (NCT03672539). Gemtuzumab ozogamicin is an antibody-drug conjugate consisting of a monoclonal antibody targeting CD33 linked to a cytotoxic derivative of calicheamicin. Free calicheamicin exerts its effect into the nucleus, where it binds DNA and induces double strand breaks, leading to cell cycle arrest and apoptosis. In principle, stem cells and non-hematopoietic cells should not be affected by this agent due to lack of CD33 expression [164]. The clinical trial aims at evaluating if giving CPX-351 and gemtuzumab ozogamicin may work better in treating patients with AML, compared to giving only one of these therapies alone. Another formula in a clinical trial is the combination of CPX-351 and ruxolitinib for the treatment of patients with secondary AML (NCT03878199). Ruxolitinib is a potent selective ATP-competitive inhibitor of Janus-associated kinases (JAK1 and JAK2), with potential antineoplastic and immunomodulating activities. More in detail, this drug may lead to a reduction in inflammation and an inhibition of cellular proliferation due to a decrease in the amount of circulating inflammatory cytokines [165]. Giving ruxolitinib and CPX-351 in combination may work better in treating patients with secondary acute myeloid leukemia compared to CPX-351 alone. Another drug currently under evaluation in combination with CPX-351 is Palbociclib, a CDK4/6 inhibitor already approved for the treatment of breast cancer [166]. Palbociclib may stop the growth of cancer cells by blocking some of the enzymes needed for cell growth. The purpose of the NCT03844997 clinical trial is to test the safety and efficacy of the combination of palbociclib and CPX-351 in patients with acute myeloid leukemia. Other drugs/agent in phase testing in combination with CPX-351 are Venetoclax, a BCL-2 inhibitor with antineoplastic activity [167], or Enasidenib, an inhibitor of isocitrate dehydrogenase 2 (IDH2), approved in the United States for the treatment of adult patients with mutant-*IDH2* relapsed or refractory (R/R) AML [168].

**Figure 5:**
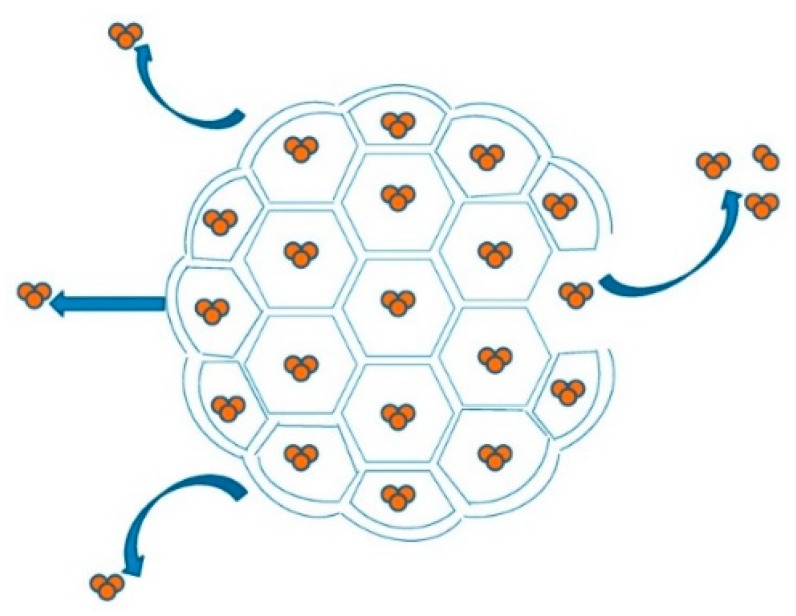
Spheroid and granular structure of a DepoFoam™ particle. Reproduced with permission from [26] (published by MDPI), licened under CC BY.

Another nanosized system currently in clinical trials for various types of leukemia, including acute myeloid leukemia (AML), acute lymphocytic leukemia (ALL), and chronic myelogenous leukemia (CML), is DepoCyt^®^ (Pacira Pharmaceuticals, Parsippany-Troy Hills, New Jersey, US) [169]. DepoCyt^®^, also known as Ara-C, is a liposomal formulation containing 10 mg/mL of cytarabine, approved in 2007 by FDA to treat lymphomatous meningitis, a life-threatening complication of lymphoma. It is administered directly intratechally to overcome blood–brain barrier crossing issues, and in comparison to free cytarabine it results in a significantly extended half-life, prolonged exposure to the therapy, and more uniform distribution. The liposomal formulation of DepoCyt^®^ exploits the DepoFoam™ drug delivery technology [170]. DepoFoam™ consists of microscopic spheroids (3–30 µm) with granular structure and single-layered lipid particles composed of a honeycomb of numerous nonconcentric internal aqueous chambers containing the bounded drug (Figure 5) [26].

Each particle contains various aqueous chambers, separated from the adjacent ones by bilayer membranes composed of lipids and phospholipids (Cholesterol: Triolein: DOPC: DPPG in 11:1:7:1 molar ratio) [171]. Upon administration of the particles, DepoFoam^TM^ technology releases the drug over a period of hours to weeks following erosion and/or reorganization of the lipid membranes. In the clinical trials here reported DepoCyt^®^ is tested in combination with either Rituximab (NCT01859819) or Obinutuzumab or Ifosfamide, Carboplatin, Etoposide (ICE) (NCT02393157). Rituximab is a chimeric monoclonal antibody that modulates the CD20 receptor, an antigen found on the surface of normal and malignant B cells, thereby inducing antibody-dependent cytotoxicity, complement-dependent cytotoxicity (CDCC), and apoptosis. It was the first monoclonal antibody approved in 1997, for the treatment of cancer. It has been tested in combination with chemotherapy for the treatment of various hematological malignancies, as a synergistic effect was expected [172]. Obinutuzumab is another monoclonal antibody directed against CD20, but differently from Rituxumab, it is a type II monoclonal antibody working primarily by inducing direct cell death and antibody-dependent cell-mediated cytotoxicity [173]. ICE chemotherapy, instead, refers to a therapeutic regimen including Ifosfamide, an alkylating antineoplastic agent, Carboplatin, a platinum-based antineoplastic drug, and Etoposide, a topoisomerase inhibitor, used in the salvage treatment of relapsed or refractory non-Hodgkin’s lymphoma and Hodgkin lymphoma. In the clinical trial in question ICE regimen has been associated with Obinutuzumab, while DepoCyt^®^ has been administered for both prophylaxis and treatment of CNS disease in order to improve the clinical outcome.

An innovative nanosized formulation, currently tested as a single agent administration for the treatment of AML, is the liposomal annamycin (NCT03315039). Annamycin is a new generation anthracycline that circumvents multidrug-resistance transporters, including p-glycoprotein, and has little to no cardiac toxicity [174]. This drug targets topoisomerase II, causing strand breaks in DNA. Its encapsulation into bilamellar liposomes composed of DMPC and DMPG in a 7:3 molar ratio results in higher entrapment of the drug and increased delivery of the drug to the tumor [89].

Marquibo^®^ (Spectrum Pharmaceuticals, Henderson, Nevada, USA), a liposomal formulation of Vincristine Sulfate, was developed in order to prolong the drug plasma circulation in comparison to free Vincristine [175]. It is composed of 0.16 mg/mL of Vincristine entrapped in an aqueous interior core of sphingomyelin/cholesterol liposomes called optisomes (sphingomyelin:cholesterol molar ratio is 60:40) [175]. The lipidic nature of these components, together with the small diameter of the resulting nanoparticles (around 100 nm), result in reduced protein binding and consequent prolonged half-life time. Vincristine is an antitumor vinca alkaloid, whose mechanism of action is related to the inhibition of microtubule formation in the mitotic spindle, resulting in an arrest of dividing cells at the metaphase stage. FDA has approved it for the treatment of relapsed ALL in adults. However, the clinical trial NCT02337478, focused on how well vincristine sulfate liposome works in treating patients with AML that has returned after a period of improvement or has not responded to previous treatment, was stopped early due to futility. Other studies, still ongoing, concern the combination of Marquibo^®^ with various agents. Among them, we can cite Venetoclax, a BCL-2 inhibitor with antineoplastic activity, Rituximab, and Bendamustine, a bifunctional mechlorethamine derivative with alkylator and antimetabolite activities: its metabolites alkylate and crosslink macromolecules, resulting in DNA, RNA, and protein synthesis inhibition, and, subsequently, apoptosis. Another promising association under evaluation is the administration of Marquibo^®^ together with inotuzumab ozogamicin (NCT03851081). Inotuzumab ozogamicin is a humanized monoclonal antibody against CD22 antigen conjugated to a cytotoxic derivative of calicheamicin, approved in several countries—including in the USA, EU, and Japan—as monotherapy for the treatment of adults with relapsed/refractory B-ALL. This construct allows the specific delivery of the cytotoxic drug to CD22 positive B cells. Giving inotuzumab ozogamicin and vincristine sulfate liposome together may work better in treating patients with CD22+ B-ALL compared to giving inotuzumab ozogamicin or vincristine sulfate liposome alone. Other possible drug/agent associations to Marquibo^®^ are reported in Table 2.

An extremely original nanosystem is BP1001, a liposome-incorporated antisense oligodeoxynucleotide that blocks the growth factor receptor-bound protein 2 (Grb2) expression [176,177]. Inhibition of Grb2 used by oncogenic tyrosine kinases for signal transduction is expected to suppress the activation of mitogen-activated protein kinase (MAPK) 1 and MAPK3 (ERK2 and ERK1) and inhibit leukemia progression. In the clinical trial NCT02923986, BP1001 is administered in association with Dasatinib, an orally bioavailable synthetic small molecule inhibitor of protein kinases, used to treat CML. Researchers hope that the combination of BP1001 and Dasatinib will provide a benefit to leukemia affected patients.

A newly developed liposomal formulation containing mitoxantrone hydrochloride is also under evaluation as a single agent administration for the treatment of Relapsed or Refractory Peripheral T-cell and NK/T-cell lymphoma [178]. Mitoxantrone is a synthetic anthracenedione anticancer drug, which is effective on lymphoma, leukemia, and other solid tumors. Its mechanism of action lies in the intercalation into DNA, causing crosslinks and strand breaks, interference with RNA and inhibition of topoisomerase II. It is cytotoxic both for proliferating and non-proliferating cells. Mitoxantrone encapsulation into liposomes has been proven to prolong its blood half-life time, improving safety and efficacy [179]. The membrane of these small unilamellar vesicles (~60 nm) is composed of hydrogenated soy phosphatidylcholine (PC), cholesterol, and pegylated lipid (DSPE-PEG).

Finally, AZD2811, a nanoparticle suspension containing Barasertib, is currently in a clinical trial for the treatment of AML. Barasertib is the prodrug of the pyrazoloquinazoline Aurora kinase B inhibitor. Aurora kinase B is overexpressed in tumor cells; its inhibition results in the disruption of spindle checkpoint functions and chromosome alignment, thus affecting cell division and proliferation and selectively inducing apoptosis [180]. Targeting Aurora B kinase with AZD2811 nanoparticles is a novel approach to deliver a cell-cycle inhibitor in AML and has a potential to improve the clinical activity seen with cell-cycle agents in this disease. In this system, the drug is encapsulated into nanosystems, known as accurins, composed of block copolymers of poly-d,l-lactide (PLA) and poly(ethylene glycol) (PEG)—both clinically validated biomaterials with a long history of safe use in humans [181]. Various accurins formulations have been tested during preclinical assays, in order to maximize the Barasertib encapsulation efficiency and the control of drug release. Finally, thanks to the improved preclinical efficacy and tolerability data displayed by the pamoic acid lead formulation, it has been selected for clinical evaluation [182].

## 7. Conclusions

Currently, the treatment of leukemia appears to be one of the most important medical areas of testing at chemical level nanosized systems for drug delivery. The investigated systems that entered the clinical trials are essentially represented by liposomes as they provide a prolonged lifetime of the drug in the systemic circulation coupled with a slow steady release of their content. The latter property is particularly important in the treatment of leukemia as the combination of drugs having different effects on the targeted cells is of paramount importance. It is well established that the maintenance of proper molecular ratios between the administrated drugs at the site of action is crucial for the success of the therapy. The slow release of the payload from the liposomal carriers guarantees the availability of the administered drugs in the proper, intended ratio. However, one may expect the other systems will provide peculiar properties that may further improve the treatment of leukemia. Certainly, the introduction of targeting vectors that selectively deliver the nanosized system to the selected cell is a route that needs further development. Then it appears that a combination strategy with some newly approved agents (such as kinase inhibitors or BCL2 inhibitors) and selective targeting by functionalized nanoparticles may open up important opportunities to cure more patients suffering from AML, ALL, and CLL.

## Figures and Tables

**Table 1:** FDA and European Medicines Agency (EMA)-approved nano-based formulations for cancer related therapy (ref: ema.europa.eu; drugs.com; fda.gov)

Brand Name	Nanotechnology	Drug	Indications	Approval
Abraxane	Albumin-Nanoparticles	Paclitaxel	Breast cancer	2005
			Non-small-cell lung carcinoma	2012
			Pancreatic cancer	2013
DaunoXome^®^	Liposome	Daunorubicin citrate	Kaposi’s sarcoma	1996
DepoCyt^®^	Liposome	Cytarabine	Neoplastic meningitis	1999
Doxil^®^/Caelyx^TM^	PEGylated Liposome	Doxorubicin hydrochloride	Kaposi’s sarcoma	1995
			Multiple myeloma	2004
			ovarian cancer	2005
Eligard^®^	PLGA [poly(lactic-co-glycolic acid)]	Leuprolide acetate	Prostate cancer	2002
Marqibo^®^	Liposome	Vincristine sulfate	Acute lymphoblastic leukemia	2012
Mepact^®^	Liposomal	Mifamurtide	Osteosarcoma	2009
Myocet^®^	Liposome	Doxorubicin	Metastatic breast cancer	2000
Nanotherm^®^	Iron oxide NPs	n.a.	Glioblastoma	2010
Onivyde^®^	Liposome	Irinotecan	Pancreatic cancer	2015

**Table 2:** Ongoing clinical trials involving nanosized systems administered alone or in combination with other drugs or biological agents for the treatment of various myeloid and lymphoid malignancies. ^1^ Trial ID refers to Clinicaltrials.gov ID.

NANOSYSTEM	ENCAPSULATED DRUG	SINGLE/ COMBINATION	DISEASE	PHASE	FIRST/ LAST POSTED	NOTES	REF	TRIAL ID^1^
Liposome	Annamycin	Single	AML	Phase I/II	2017/2019		[Gil et al., 2019]	NCT03315039
Liposome	Cytarabine (L-ARA-C, Depocyt^®^)	Rituximab	Lymphoma	Phase II	2013–2019		[Jurczak et al., 2015]	NCT01859819
Liposome	Cytarabine (L-ARA-C, Depocyt^®^)	Obinutuzumab or Ifosfamide, Carboplatin, Etoposide (ICE)	Lymphoma	Phase II	2015–2019		[Jurczak et al., 2015]	NCT02393157
Liposome	Daunorubicin-Cytarabine (CPX-351, Vyxeos^®^)	Single	Refractory AML	Phase II	2019		[Mayer et al., Chen 2018 et al.]	NCT04049539
Liposome	Daunorubicin-Cytarabine (CPX-351, Vyxeos^®^)	Single	ALL, Refractory ALL, Recurrent ALL	Phase II	2018/2019		[Mayer et al., Chen 2018 et al.]	NCT03575325
Liposome	Daunorubicin-Cytarabine (CPX-351, Vyxeos^®^)	Enasidenib	Recurrent AML	Phase II	2019		[Mayer et al., Chen 2018 et al.]	NCT03825796
Liposome	Daunorubicin-Cytarabine (CPX-351, Vyxeos)	Gemtuzumab Ozogamicin	AML, CML, Recurrent AML, Refractory AML	Phase II	2018/2019		[Mayer et al., Chen 2018 et al., Baron et al.]	NCT03672539
Liposome	Daunorubicin-Cytarabine (CPX-351, Vyxeos^®^)	Palbociclib	AML	Phase I/II	2019		[Mayer et al., Chen 2018 et al., Winer et al., 2019]	NCT03844997
Liposome	Daunorubicin-Cytarabine (CPX-351, Vyxeos^®^)	Ruxolitinib	AML, ALL	Phase I/II	2019		[Mayer et al., Chen 2018 et al., Eghtedar et al., 2012]	NCT03878199
Liposome	Daunorubicin-Cytarabine (CPX-351, Vyxeos^®^)	Venetoclax	AML	Phase Ib	2019	CPX-351 Lower Intensity Therapy (LIT)	[Mayer et al., Chen 2018 et al., Massimino et al., 2018]	NCT04038437
Liposome	Daunorubicin-Cytarabine (CPX-351, Vyxeos^®^)	Venetoclax	AML	Phase II	2018/2019		[Mayer et al., Chen 2018 et al., Massimino et al., 2018]]	NCT03629171
Liposome	Daunorubicin-Cytarabine (CPX-351, Vyxeos^®^)	Venetoclax or Midostaurin or Enasidenib	AML	Phase I	2019		[Mayer et al., Chen 2018 et al., Massimino et al., 2018]]	NCT04075747
Liposome	Grb2 Antisense Oligonucleotide (BP1001)	Dasatinib	AML, CML	Phase I/II	2016–2019		[Thomas et al., 2018; Ohanian et al., 2018]	NCT02923986
Liposome	Mitoxantrone Hydrochloride	Single	Peripheral T-cell and NK/T-cell Lymphoma	Phase II	2018		[Huang et al. 2019]	NCT03776279
Liposome	Vincristine	Venetoclax	ALL	Phase I/II	2018–2019		[Pathak et al., 2014]	NCT03504644
Liposome	Vincristine sulfate (Marquibo^®^)	Single	AML	Phase II	2015/2019	The study was stopped early due to futility	[Shah et al., 2016]	NCT02337478
Liposome	Vincristine sulfate (Marquibo^®^)	Bortezomib, Clofarabine, Cyclophosphamide, Dexamethasone, Etoposide, Ofatumumab, Pegfilgrastim, Rituximab	ALL, Burkitt Leukemia, Burkitt Lymphoma	Phase II	2017–2019		[Shah et al., 2016]	NCT03136146
Liposome	Vincristine sulfate (Marquibo^®^)	Dexamethasone, Mitoxantrone and Asparaginase (UK ALL R3)	ALL	Phase I	2016–2019		[Shah et al., 2016]	NCT02879643
Liposome	Vincristine sulfate (Marquibo^®^)	Inotuzumab Ozogamicin	ALL	Phase I/II	2019		[Shah et al., 2016, Al-Salama ZT 2018]	NCT03851081
Liposome	Vincristine sulfate (Marquibo^®^)	Rituximab Bendamustine	Indolent B cell Lymphoma	Phase I	2014–2019		[Shah et al., 2016]	NCT02257242
Nanoparticle	AZD2811 (Barasertib)	Azacitidine	AML	Phase I/II	2017–2019		[Floc’h et al., 2017]	NCT03217838

## Abbreviations

ALL	Acute lymphoblastic leukemia
AML	Acute myeloid leukemia
AML-MRC	AML with myelodysplastic related changes
APL	Acute promyelocytic leukemia
ATRA	All-trans-retinoic acid
BCR	Breakpoint cluster region
CLL	Chronic lymphocytic leukemia
CMC	Critical micelle concentration
CML	Chronic myeloid leukemia
DMPG	Dimyristoyl phosphatidylglycerol
DOPC	Dioleoyl phosphatidylcholine
DOPE	Dioleoyl phosphatidylethanolamine
DOPE	Dioleoylphosphatidyl ethanolamine
DOTAP	1,2-dioleoyl-3-trimethylammonium-propane
DPPC	Dipalmitoyl phosphatidylcholine
DPPG	Dipalmitoyl phosphatidylglycerol
EMA	European Medicines Agency
EPR	Enhanced permeability and retention
FDA	Food and Drug Administration
FLT3	Fms-related tyrosine kinase 3
LSC	Leukemic stem cell
MRI	Magnetic resonance imaging
NLCs	Nanostructured lipid carriers
OA	Oleic acid
OS	Overall survival
PDGF-Rs	Receptors for platelet-derived growth factor
PEG	Polyethylene glycol
PGA	Poly glycolic acid
PLA	Poly lactic acid
PLGA	Poly(lactic-co-glycolic acid)
PTL	Parthenolide
RES	Reticuloendothelial system
RGD	Arginylglycylaspartic acid
ROR1	Tyrosine-protein kinase transmembrane receptor
SLNs	Solid lipid nanoparticles
SYK	Spleen tyrosine kinase
t-AML	Therapy-related AML
TKIs	Tyrosine kinase inhibitors
T_m_	Phase transition temperature
VEGFRs	Vascular endothelial growth factor receptors
VSLI	Vincristine sulfate liposome injection

## References

[1] Miranda-Filho A., Pineros M., Ferlay J., Soerjomataram I., Monnereau A., Bray F. (2018). Epidemiological patterns of leukaemia in 184 countries: A population-based study. Lancet Haematol..

[2] Torre L.A., Siegel R.L., Ward E.M., Jemal A. (2016). Global Cancer Incidence and Mortality Rates and Trends--An Update. Cancer Epidemiol. Biomark. Prev..

[3] Taylor J., Xiao W., Abdel-Wahab O. (2017). Diagnosis and classification of hematologic malignancies on the basis of genetics. Blood.

[4] Hu R., Wu Y., Jiang X., Zhang W., Xu L. (2011). Clinical symptoms and chemotherapy completion in elderly patients with newly diagnosed acute leukemia: A retrospective comparison study with a younger cohort. BMC Cancer.

[5] Krause D.S., Van Etten R.A. (2007). Right on target: Eradicating leukemic stem cells. Trends Mol. Med..

[6] Rothenberg-Thurley M., Amler S., Goerlich D., Kohnke T., Konstandin N.P., Schneider S., Sauerland M.C., Herold T., Hubmann M., Ksienzyk B. (2018). Persistence of pre-leukemic clones during first remission and risk of relapse in acute myeloid leukemia. Leukemia.

[7] Perl A.E. (2017). The role of targeted therapy in the management of patients with AML. Blood Adv..

[8] Jabbour E., Cortes J.E., Ghanem H., O’Brien S., Kantarjian H.M. (2008). Targeted therapy in chronic myeloid leukemia. Expert Rev. Anticancer Ther..

[9] Bae K.H., Chung H.J., Park T.G. (2011). Nanomaterials for cancer therapy and imaging. Mol. Cells.

[10] (2009). ‘Plenty of room’ revisited. Nat. Nanotechnol..

[11] Fornaguera C., Garcia-Celma M.J. (2017). Personalized Nanomedicine: A Revolution at the Nanoscale. J. Pers. Med..

[12] Bangham A.D., Horne R.W. (1964). Negative Staining of Phospholipids and Their Structural Modification by Surface-Active Agents as Observed in the Electron Microscope. J. Mol. Biol..

[13] Patra J.K., Das G., Fraceto L.F., Campos E.V.R., Rodriguez-Torres M.D.P., Acosta-Torres L.S., Diaz-Torres L.A., Grillo R., Swamy M.K., Sharma S. (2018). Nano based drug delivery systems: recent developments and future prospects. J. Nanobiotechnol..

[14] Hua S., de Matos M.B.C., Metselaar J.M., Storm G. (2018). Current Trends and Challenges in the Clinical Translation of Nanoparticulate Nanomedicines: Pathways for Translational Development and Commercialization. Front. Pharmacol..

[15] Gubernator J. (2011). Active methods of drug loading into liposomes: Recent strategies for stable drug entrapment and increased in vivo activity. Expert Opin. Drug Deliv..

[16] Navya P.N., Kaphle A., Srinivas S.P., Bhargava S.K., Rotello V.M., Daima H.K. (2019). Current trends and challenges in cancer management and therapy using designer nanomaterials. Nano Converg..

[17] Singh A.P., Biswas A., Shukla A., Maiti P. (2019). Targeted therapy in chronic diseases using nanomaterial-based drug delivery vehicles. Signal. Transduct Target. Ther..

[18] Coukell A.J., Brogden R.N. (1998). Liposomal amphotericin B. Therapeutic use in the management of fungal infections and visceral leishmaniasis. Drugs.

[19] Adler-Moore J., Proffitt R.T. (2002). AmBisome: Liposomal formulation, structure, mechanism of action and pre-clinical experience. J. Antimicrob. Chemother..

[20] Fluhmann B., Ntai I., Borchard G., Simoens S., Muhlebach S. (2019). Nanomedicines: The magic bullets reaching their target?. Eur. J. Pharm. Sci..

[21] Bozzuto G., Molinari A. (2015). Liposomes as nanomedical devices. Int. J. Nanomed..

[22] Akbarzadeh A., Rezaei-Sadabady R., Davaran S., Joo S.W., Zarghami N., Hanifehpour Y., Samiei M., Kouhi M., Nejati-Koshki K. (2013). Liposome: Classification, preparation, and applications. Nanoscale Res. Lett..

[23] Sercombe L., Veerati T., Moheimani F., Wu S.Y., Sood A.K., Hua S. (2015). Advances and Challenges of Liposome Assisted Drug Delivery. Front. Pharmacol..

[24] Allen T.M. (1998). Liposomal drug formulations. Rationale for development and what we can expect for the future. Drugs.

[25] Daraee H., Etemadi A., Kouhi M., Alimirzalu S., Akbarzadeh A. (2016). Application of liposomes in medicine and drug delivery. Artif. Cells Nanomed. Biotechnol..

[26] Bulbake U., Doppalapudi S., Kommineni N., Khan W. (2017). Liposomal Formulations in Clinical Use: An Updated Review. Pharmaceutics.

[27] Zhao W., Zhuang S., Qi X.R. (2011). Comparative study of the in vitro and in vivo characteristics of cationic and neutral liposomes. Int. J. Nanomed..

[28] Lombardo D., Calandra P., Barreca D., Magazu S., Kiselev M.A. (2016). Soft Interaction in Liposome Nanocarriers for Therapeutic Drug Delivery. Nanomaterials.

[29] Felgner P.L., Ringold G.M. (1989). Cationic liposome-mediated transfection. Nature.

[30] Pedroso de Lima M.C., Neves S., Filipe A., Duzgunes N., Simoes S. (2003). Cationic liposomes for gene delivery: From biophysics to biological applications. Curr. Med. Chem..

[31] Briuglia M.L., Rotella C., McFarlane A., Lamprou D.A. (2015). Influence of cholesterol on liposome stability and on in vitro drug release. Drug Deliv. Transl. Res..

[32] Immordino M.L., Dosio F., Cattel L. (2006). Stealth liposomes: Review of the basic science, rationale, and clinical applications, existing and potential. Int. J. Nanomed..

[33] Chen E.C., Fathi A.T., Brunner A.M. (2018). Reformulating acute myeloid leukemia: Liposomal cytarabine and daunorubicin (CPX-351) as an emerging therapy for secondary AML. Onco Targets Ther..

[34] Garello F., Terreno E. (2018). Sonosensitive MRI Nanosystems as Cancer Theranostics: A Recent Update. Front. Chem..

[35] Kneidl B., Peller M., Winter G., Lindner L.H., Hossann M. (2014). Thermosensitive liposomal drug delivery systems: state of the art review. Int. J. Nanomed..

[36] Besse H.C., Barten-van Rijbroek A.D., van der Wurff-Jacobs K.M.G., Bos C., Moonen C.T.W., Deckers R. (2019). Tumor Drug Distribution after Local Drug Delivery by Hyperthermia, In Vivo. Cancers.

[37] Hanafy N.A.N., El-Kemary M., Leporatti S. (2018). Micelles Structure Development as a Strategy to Improve Smart Cancer Therapy. Cancers.

[38] Soleimani Zohr Shiri M., Henderson W., Mucalo M.R. (2019). A Review of The Lesser-Studied Microemulsion-Based Synthesis Methodologies Used for Preparing Nanoparticle Systems of The Noble Metals, Os, Re, Ir and Rh. Materials.

[39] Martinelli C., Pucci C., Ciofani G. (2019). Nanostructured carriers as innovative tools for cancer diagnosis and therapy. APL Bioeng.

[40] Lu Y., Zhang E., Yang J., Cao Z. (2018). Strategies to improve micelle stability for drug delivery. Nano Res..

[41] Biswas S., Kumari P., Lakhani P.M., Ghosh B. (2016). Recent advances in polymeric micelles for anti-cancer drug delivery. Eur. J. Pharm. Sci..

[42] Nasir A., Kausar A., Younus A. (2015). A Review on Preparation, Properties and Applications of Polymeric Nanoparticle-Based Materials. Polym.-Plast. Technol..

[43] Kamaly N., Yameen B., Wu J., Farokhzad O.C. (2016). Degradable Controlled-Release Polymers and Polymeric Nanoparticles: Mechanisms of Controlling Drug Release. Chem. Rev..

[44] Makadia H.K., Siegel S.J. (2011). Poly Lactic-co-Glycolic Acid (PLGA) as Biodegradable Controlled Drug Delivery Carrier. Polymers.

[45] Sadat Tabatabaei Mirakabad F., Nejati-Koshki K., Akbarzadeh A., Yamchi M.R., Milani M., Zarghami N., Zeighamian V., Rahimzadeh A., Alimohammadi S., Hanifehpour Y. (2014). PLGA-based nanoparticles as cancer drug delivery systems. Asian Pac. J. Cancer Prev..

[46] Dordunoo S.K., Jackson J.K., Arsenault L.A., Oktaba A.M., Hunter W.L., Burt H.M. (1995). Taxol encapsulation in poly(epsilon-caprolactone) microspheres. Cancer Chemother. Pharmacol..

[47] Najlaoui F., Pigeon P., Aroui S., Pezet M., Sancey L., Marrakchi N., Rhouma A., Jaouen G., De Waard M., Busser B. (2018). Anticancer properties of lipid and poly(epsilon-caprolactone) nanocapsules loaded with ferrocenyl-tamoxifen derivatives. J. Pharm. Pharmacol..

[48] Zhang H., Wu F., Li Y., Yang X., Huang J., Lv T., Zhang Y., Chen J., Chen H., Gao Y. (2016). Chitosan-based nanoparticles for improved anticancer efficacy and bioavailability of mifepristone. Beilstein J. Nanotechnol..

[49] Kamath P.R., Sunil D. (2017). Nano-Chitosan Particles in Anticancer Drug Delivery: An Up-to-Date Review. Mini. Rev. Med. Chem..

[50] Mohammed M.A., Syeda J.T.M., Wasan K.M., Wasan E.K. (2017). An Overview of Chitosan Nanoparticles and Its Application in Non-Parenteral Drug Delivery. Pharmaceutics.

[51] Hajebi S., Rabiee N., Bagherzadeh M., Ahmadi S., Rabiee M., Roghani-Mamaqani H., Tahriri M., Tayebi L., Hamblin M.R. (2019). Stimulus-responsive polymeric nanogels as smart drug delivery systems. Acta Biomater..

[52] Zhang Q., Colazo J., Berg D., Mugo S.M., Serpe M.J. (2017). Multiresponsive Nanogels for Targeted Anticancer Drug Delivery. Mol. Pharm..

[53] Soni G., Yadav K.S. (2016). Nanogels as potential nanomedicine carrier for treatment of cancer: A mini review of the state of the art. Saudi. Pharm. J..

[54] Wissing S.A., Kayser O., Muller R.H. (2004). Solid lipid nanoparticles for parenteral drug delivery. Adv. Drug Deliv. Rev..

[55] Liu J., Xiao Y., Allen C. (2004). Polymer-drug compatibility: A guide to the development of delivery systems for the anticancer agent, ellipticine. J. Pharm. Sci..

[56] Ghasemiyeh P., Mohammadi-Samani S. (2018). Solid lipid nanoparticles and nanostructured lipid carriers as novel drug delivery systems: Applications, advantages and disadvantages. Res. Pharm. Sci..

[57] Muller R.H., Radtke M., Wissing S.A. (2002). Nanostructured lipid matrices for improved microencapsulation of drugs. Int. J. Pharm..

[58] Wong H.L., Bendayan R., Rauth A.M., Li Y., Wu X.Y. (2007). Chemotherapy with anticancer drugs encapsulated in solid lipid nanoparticles. Adv. Drug Deliv. Rev..

[59] Serpe L., Catalano M.G., Cavalli R., Ugazio E., Bosco O., Canaparo R., Muntoni E., Frairia R., Gasco M.R., Eandi M. (2004). Cytotoxicity of anticancer drugs incorporated in solid lipid nanoparticles on HT-29 colorectal cancer cell line. Eur. J. Pharm. Biopharm..

[60] Malam Y., Loizidou M., Seifalian A.M. (2009). Liposomes and nanoparticles: Nanosized vehicles for drug delivery in cancer. Trends Pharmacol. Sci..

[61] Anselmo A.C., Mitragotri S. (2015). A Review of Clinical Translation of Inorganic Nanoparticles. AAPS J..

[62] Farooq M.U., Novosad V., Rozhkova E.A., Wali H., Ali A., Fateh A.A., Neogi P.B., Neogi A., Wang Z. (2018). Gold Nanoparticles-enabled Efficient Dual Delivery of Anticancer Therapeutics to HeLa Cells. Sci. Rep..

[63] Richards D.A., Maruani A., Chudasama V. (2017). Antibody fragments as nanoparticle targeting ligands: a step in the right direction. Chem. Sci..

[64] Feng Q., Liu Y., Huang J., Chen K., Huang J., Xiao K. (2018). Uptake, distribution, clearance, and toxicity of iron oxide nanoparticles with different sizes and coatings. Sci. Rep..

[65] Vangijzegem T., Stanicki D., Laurent S. (2019). Magnetic iron oxide nanoparticles for drug delivery: Applications and characteristics. Expert Opin. Drug Deliv..

[66] Bharti C., Nagaich U., Pal A.K., Gulati N. (2015). Mesoporous silica nanoparticles in target drug delivery system: A review. Int. J. Pharm. Investig..

[67] Shahbazi M.A., Herranz B., Santos H.A. (2012). Nanostructured porous Si-based nanoparticles for targeted drug delivery. Biomatter.

[68] Yang Y., Yu C. (2016). Advances in silica based nanoparticles for targeted cancer therapy. Nanomedicine.

[69] Fang M., Peng C.W., Pang D.W., Li Y. (2012). Quantum dots for cancer research: Current status, remaining issues, and future perspectives. Cancer Biol. Med..

[70] Bosch F., Rosich L. (2008). The contributions of Paul Ehrlich to pharmacology: A tribute on the occasion of the centenary of his Nobel Prize. Pharmacology.

[71] Singh R.K., Kumar S., Prasad D.N., Bhardwaj T.R. (2018). Therapeutic journery of nitrogen mustard as alkylating anticancer agents: Historic to future perspectives. Eur. J. Med. Chem..

[72] Farber S., Diamond L.K. (1948). Temporary remissions in acute leukemia in children produced by folic acid antagonist, 4-aminopteroyl-glutamic acid. N. Engl. J. Med..

[73] Strebhardt K., Ullrich A. (2008). Paul Ehrlich’s magic bullet concept: 100 years of progress. Nat. Rev. Cancer.

[74] Baselga J. (2006). Targeting tyrosine kinases in cancer: The second wave. Science.

[75] Moen M.D., McKeage K., Plosker G.L., Siddiqui M.A. (2007). Imatinib: A review of its use in chronic myeloid leukaemia. Drugs.

[76] Papaetis G.S., Syrigos K.N. (2009). Sunitinib: A multitargeted receptor tyrosine kinase inhibitor in the era of molecular cancer therapies. BioDrugs.

[77] Kohler G., Milstein C. (1975). Continuous cultures of fused cells secreting antibody of predefined specificity. Nature.

[78] Marks L. (2012). The birth pangs of monoclonal antibody therapeutics: The failure and legacy of Centoxin. MAbs.

[79] Liu J.K. (2014). The history of monoclonal antibody development - Progress, remaining challenges and future innovations. Ann. Med. Surg. (Lond.).

[80] Carter P. (2001). Improving the efficacy of antibody-based cancer therapies. Nat. Rev. Cancer.

[81] Schrama D., Reisfeld R.A., Becker J.C. (2006). Antibody targeted drugs as cancer therapeutics. Nat. Rev. Drug Discov..

[82] Vu T., Claret F.X. (2012). Trastuzumab: Updated mechanisms of action and resistance in breast cancer. Front. Oncol..

[83] Pierpont T.M., Limper C.B., Richards K.L. (2018). Past, Present, and Future of Rituximab-The World’s First Oncology Monoclonal Antibody Therapy. Front. Oncol..

[84] Arachchige M.C., Reshetnyak Y.K., Andreev O.A. (2016). Corrigendum to “Advanced targeted nanomedicine” [J. Biotechnol. 202 (2015) 88-97]. J. Biotechnol..

[85] Nie S. (2010). Understanding and overcoming major barriers in cancer nanomedicine. Nanomedicine.

[86] Maeda H., Wu J., Sawa T., Matsumura Y., Hori K. (2000). Tumor vascular permeability and the EPR effect in macromolecular therapeutics: A review. J. Control. Release.

[87] Stockhofe K., Postema J.M., Schieferstein H., Ross T.L. (2014). Radiolabeling of Nanoparticles and Polymers for PET Imaging. Pharmaceuticals.

[88] Misra R., Acharya S., Sahoo S.K. (2010). Cancer nanotechnology: Application of nanotechnology in cancer therapy. Drug Discov. Today.

[89] Rosenblum D., Joshi N., Tao W., Karp J.M., Peer D. (2018). Progress and challenges towards targeted delivery of cancer therapeutics. Nat. Commun..

[90] Vinhas R., Mendes R., Fernandes A.R., Baptista P.V. (2017). Nanoparticles-Emerging Potential for Managing Leukemia and Lymphoma. Front. Bioeng. Biotechnol..

[91] Barui A.K., Oh J.Y., Jana B., Kim C., Ryu J.-H. (2019). Cancer-Targeted Nanomedicine: Overcoming the Barrier of the Protein Corona. Adv. Ther. N/A.

[92] Friedman A.D., Claypool S.E., Liu R. (2013). The smart targeting of nanoparticles. Curr. Pharm. Des..

[93] Wang X., Yang L., Chen Z.G., Shin D.M. (2008). Application of nanotechnology in cancer therapy and imaging. CA Cancer J. Clin..

[94] Attarwala H. (2010). Role of antibodies in cancer targeting. J. Nat. Sci. Biol. Med..

[95] Wang D., Sun Y., Liu Y., Meng F., Lee R.J. (2018). Clinical translation of immunoliposomes for cancer therapy: Recent perspectives. Expert Opin. Drug Deliv..

[96] Kontermann R.E. (2006). Immunoliposomes for cancer therapy. Curr. Opin. Mol. Ther..

[97] Frejd F.Y., Kim K.T. (2017). Affibody molecules as engineered protein drugs. Exp. Mol. Med..

[98] Bi Y., Hao F., Yan G., Teng L., Lee R.J., Xie J. (2016). Actively Targeted Nanoparticles for Drug Delivery to Tumor. Curr. Drug Metab..

[99] Gu F.X., Karnik R., Wang A.Z., Alexis F., Levy-Nissenbaum E., Hong S., Langer R.S., Farokhzad O.C. (2007). Targeted nanoparticles for cancer therapy. Nano Today.

[100] Nieberler M., Reuning U., Reichart F., Notni J., Wester H.J., Schwaiger M., Weinmuller M., Rader A., Steiger K., Kessler H. (2017). Exploring the Role of RGD-Recognizing Integrins in Cancer. Cancers.

[101] Mayer G. (2009). The chemical biology of aptamers. Angew. Chem. Int. Ed. Engl..

[102] Morita Y., Leslie M., Kameyama H., Volk D.E., Tanaka T. (2018). Aptamer Therapeutics in Cancer: Current and Future. Cancers.

[103] Wilhelm S., Tavares A.J., Dai Q., Ohta S., Audet J., Dvorak H.F., Chan W.C.W. (2016). Analysis of nanoparticle delivery to tumours. Nat. Rev. Mater..

[104] De Kouchkovsky I., Abdul-Hay M. (2016). ‘Acute myeloid leukemia: A comprehensive review and 2016 update’. Blood Cancer J..

[105] Dombret H., Gardin C. (2016). An update of current treatments for adult acute myeloid leukemia. Blood.

[106] Dohner H., Weisdorf D.J., Bloomfield C.D. (2015). Acute Myeloid Leukemia. N. Engl. J. Med..

[107] Lai C., Doucette K., Norsworthy K. (2019). Recent drug approvals for acute myeloid leukemia. J. Hematol. Oncol..

[108] Stein E.M., Tallman M.S. (2016). Emerging therapeutic drugs for AML. Blood.

[109] Godley L.A., Larson R.A. (2008). Therapy-related myeloid leukemia. Semin. Oncol..

[110] Vardiman J., Reichard K. (2015). Acute Myeloid Leukemia With Myelodysplasia-Related Changes. Am. J. Clin. Pathol..

[111] Mayer L.D., Tardi P., Louie A.C. (2019). CPX-351: A nanoscale liposomal co-formulation of daunorubicin and cytarabine with unique biodistribution and tumor cell uptake properties. Int. J. Nanomed..

[112] Krauss A.C., Gao X., Li L., Manning M.L., Patel P., Fu W., Janoria K.G., Gieser G., Bateman D.A., Przepiorka D. (2019). FDA Approval Summary: (Daunorubicin and Cytarabine) Liposome for Injection for the Treatment of Adults with High-Risk Acute Myeloid Leukemia. Clin. Cancer Res..

[113] Lancet J.E., Uy G.L., Cortes J.E., Newell L.F., Lin T.L., Ritchie E.K., Stuart R.K., Strickland S.A., Hogge D., Solomon S.R. (2018). CPX-351 (cytarabine and daunorubicin) Liposome for Injection Versus Conventional Cytarabine Plus Daunorubicin in Older Patients With Newly Diagnosed Secondary Acute Myeloid Leukemia. J. Clin. Oncol..

[114] Daver N., Schlenk R.F., Russell N.H., Levis M.J. (2019). Targeting FLT3 mutations in AML: Review of current knowledge and evidence. Leukemia.

[115] Small D. (2006). FLT3 mutations: Biology and treatment. Hematol. Am. Soc. Hematol. Educ. Program..

[116] Jiang X., Bugno J., Hu C., Yang Y., Herold T., Qi J., Chen P., Gurbuxani S., Arnovitz S., Strong J. (2016). Eradication of Acute Myeloid Leukemia with FLT3 Ligand-Targeted miR-150 Nanoparticles. Cancer Res..

[117] Garzon R., Heaphy C.E., Havelange V., Fabbri M., Volinia S., Tsao T., Zanesi N., Kornblau S.M., Marcucci G., Calin G.A. (2009). MicroRNA 29b functions in acute myeloid leukemia. Blood.

[118] Huang X., Schwind S., Yu B., Santhanam R., Wang H., Hoellerbauer P., Mims A., Klisovic R., Walker A.R., Chan K.K. (2013). Targeted delivery of microRNA-29b by transferrin-conjugated anionic lipopolyplex nanoparticles: A novel therapeutic strategy in acute myeloid leukemia. Clin. Cancer Res..

[119] Mahotka C., Bhatia S., Kollet J., Grinstein E. (2018). Nucleolin promotes execution of the hematopoietic stem cell gene expression program. Leukemia.

[120] Deng R., Ji B., Yu H., Bao W., Yang Z., Yu Y., Cui Y., Du Y., Song M., Liu S. (2019). Multifunctional Gold Nanoparticles Overcome MicroRNA Regulatory Network Mediated-Multidrug Resistant Leukemia. Sci. Rep..

[121] Degos L., Wang Z.Y. (2001). All trans retinoic acid in acute promyelocytic leukemia. Oncogene.

[122] Garattini E., Terao M. (2004). Atypical retinoids: An expanding series of anti-leukemia and anti-cancer agents endowed with selective apoptotic activity. J. Chemother..

[123] Garattini E., Parrella E., Diomede L., Gianni M., Kalac Y., Merlini L., Simoni D., Zanier R., Ferrara F.F., Chiarucci I. (2004). ST1926, a novel and orally active retinoid-related molecule inducing apoptosis in myeloid leukemia cells: Modulation of intracellular calcium homeostasis. Blood.

[124] El-Houjeiri L., Saad W., Hayar B., Aouad P., Tawil N., Abdel-Samad R., Hleihel R., Hamie M., Mancinelli A., Pisano C. (2017). Antitumor Effect of the Atypical Retinoid ST1926 in Acute Myeloid Leukemia and Nanoparticle Formulation Prolongs Lifespan and Reduces Tumor Burden of Xenograft Mice. Mol. Cancer Ther..

[125] Niu F., Yan J., Ma B., Li S., Shao Y., He P., Zhang W., He W., Ma P.X., Lu W. (2018). Lanthanide-doped nanoparticles conjugated with an anti-CD33 antibody and a p53-activating peptide for acute myeloid leukemia therapy. Biomaterials.

[126] Sun S., Zou H., Li L., Liu Q., Ding N., Zeng L., Li H., Mao S. (2019). CD123/CD33 dual-antibody modified liposomes effectively target acute myeloid leukemia cells and reduce antigen-negative escape. Int. J. Pharm..

[127] Houshmand M., Blanco T.M., Circosta P., Yazdi N., Kazemi A., Saglio G., Zarif M.N. (2019). Bone marrow microenvironment: The guardian of leukemia stem cells. World J. Stem. Cells.

[128] Laverdiere I., Boileau M., Neumann A.L., Frison H., Mitchell A., Ng S.W.K., Wang J.C.Y., Minden M.D., Eppert K. (2018). Leukemic stem cell signatures identify novel therapeutics targeting acute myeloid leukemia. Blood Cancer J..

[129] Ho T.C., LaMere M., Stevens B.M., Ashton J.M., Myers J.R., O’Dwyer K.M., Liesveld J.L., Mendler J.H., Guzman M., Morrissette J.D. (2016). Evolution of acute myelogenous leukemia stem cell properties after treatment and progression. Blood.

[130] Bausch-Fluck D., Hofmann A., Bock T., Frei A.P., Cerciello F., Jacobs A., Moest H., Omasits U., Gundry R.L., Yoon C. (2015). A mass spectrometric-derived cell surface protein atlas. PLoS ONE.

[131] Lin T.Y., Zhu Y., Li Y., Zhang H., Ma A.H., Long Q., Keck J., Lam K.S., Pan C.X., Jonas B.A. (2019). Daunorubicin-containing CLL1-targeting nanomicelles have anti-leukemia stem cell activity in acute myeloid leukemia. Nanomedicine.

[132] Zong H., Sen S., Zhang G., Mu C., Albayati Z.F., Gorenstein D.G., Liu X., Ferrari M., Crooks P.A., Roboz G.J. (2016). In vivo targeting of leukemia stem cells by directing parthenolide-loaded nanoparticles to the bone marrow niche. Leukemia.

[133] Baranello M.P., Bauer L., Jordan C.T., Benoit D.S.W. (2015). Micelle Delivery of Parthenolide to Acute Myeloid Leukemia Cells. Cell Mol. Bioeng..

[134] Houshmand M., Simonetti G., Circosta P., Gaidano V., Cignetti A., Martinelli G., Saglio G., Gale R.P. (2019). Chronic myeloid leukemia stem cells. Leukemia.

[135] Houshmand M., Circosta P., Saglio G. (2018). Immature CML cells implement a BMP autocrine loop to escape TKI treatment. Transl. Cancer Res..

[136] Ross D.M., Masszi T., Gomez Casares M.T., Hellmann A., Stentoft J., Conneally E., Garcia-Gutierrez V., Gattermann N., le Coutre P.D., Martino B. (2018). Durable treatment-free remission in patients with chronic myeloid leukemia in chronic phase following frontline nilotinib: 96-week update of the ENESTfreedom study. J. Cancer Res. Clin. Oncol..

[137] Liu D., Xing J., Xiong F., Yang F., Gu N. (2017). Preparation and in vivo safety evaluations of antileukemic homoharringtonine-loaded PEGylated liposomes. Drug Dev. Ind. Pharm..

[138] Yang X., Pang J., Shen N., Yan F., Wu L.C., Al-Kali A., Litzow M.R., Peng Y., Lee R.J., Liu S. (2016). Liposomal bortezomib is active against chronic myeloid leukemia by disrupting the Sp1-BCR/ABL axis. Oncotarget.

[139] Jyotsana N., Sharma A., Chaturvedi A., Budida R., Scherr M., Kuchenbauer F., Lindner R., Noyan F., Suhs K.W., Stangel M. (2019). Lipid nanoparticle-mediated siRNA delivery for safe targeting of human CML in vivo. Ann. Hematol..

[140] Terwilliger T., Abdul-Hay M. (2017). Acute lymphoblastic leukemia: A comprehensive review and 2017 update. Blood Cancer J..

[141] Paul S., Kantarjian H., Jabbour E.J. (2016). Adult Acute Lymphoblastic Leukemia. Mayo. Clin. Proc..

[142] Hunger S.P., Mullighan C.G. (2015). Acute Lymphoblastic Leukemia in Children. N. Engl. J. Med..

[143] Mohseni M., Uludag H., Brandwein J.M. (2018). Advances in biology of acute lymphoblastic leukemia (ALL) and therapeutic implications. Am. J. Blood Res..

[144] Dudeja S., Gupta S., Sharma S., Jain A., Sharma S., Jain P., Aneja S., Chandra J. (2019). Incidence of vincristine induced neurotoxicity in children with acute lymphoblastic leukemia and its correlation with nutritional deficiencies. Pediatr. Hematol. Oncol..

[145] Mora E., Smith E.M., Donohoe C., Hertz D.L. (2016). Vincristine-induced peripheral neuropathy in pediatric cancer patients. Am. J. Cancer Res..

[146] Davis T., Farag S.S. (2013). Treating relapsed or refractory Philadelphia chromosome-negative acute lymphoblastic leukemia: Liposome-encapsulated vincristine. Int. J. Nanomed..

[147] Schiller G.J., Damon L.E., Coutre S.E., Hsu P., Bhat G., Douer D. (2018). High-Dose Vincristine Sulfate Liposome Injection, for Advanced, Relapsed, or Refractory Philadelphia Chromosome-Negative Acute Lymphoblastic Leukemia in an Adolescent and Young Adult Subgroup of a Phase 2 Clinical Trial. J. Adolesc. Young Adult Oncol..

[148] Liu D., Mamorska-Dyga A. (2017). Syk inhibitors in clinical development for hematological malignancies. J. Hematol. Oncol..

[149] Uckun F.M., Qazi S. (2014). SYK as a New Therapeutic Target in B-Cell Precursor Acute Lymphoblastic Leukemia. J. Cancer Ther..

[150] Uckun F.M., Myers D.E., Cheng J., Qazi S. (2015). Liposomal Nanoparticles of a Spleen Tyrosine Kinase P-Site Inhibitor Amplify the Potency of Low Dose Total Body Irradiation Against Aggressive B-Precursor Leukemia and Yield Superior Survival Outcomes in Mice. EBioMedicine.

[151] Wang K., Wei G., Liu D. (2012). CD19: A biomarker for B cell development, lymphoma diagnosis and therapy. Exp. Hematol. Oncol..

[152] Yan J., Wolff M.J., Unternaehrer J., Mellman I., Mamula M.J. (2005). Targeting antigen to CD19 on B cells efficiently activates T cells. Int. Immunol..

[153] Myers D.E., Yiv S., Qazi S., Ma H., Cely I., Shahidzadeh A., Arellano M., Finestone E., Gaynon P.S., Termuhlen A. (2014). CD19-antigen specific nanoscale liposomal formulation of a SYK P-site inhibitor causes apoptotic destruction of human B-precursor leukemia cells. Integr. Biol..

[154] Zhang J., Tang Y., Li S., Liao C., Guo X. (2010). Targeting of the B-lineage leukemia stem cells and their progeny with norcantharidin encapsulated liposomes modified with a novel CD19 monoclonal antibody 2E8 in vitro. J. Drug Target..

[155] Hallek M. (2019). Chronic lymphocytic leukemia: 2020 update on diagnosis, risk stratification and treatment. Am. J. Hematol..

[156] Jain N., Thompson P., Ferrajoli A., Nabhan C., Mato A.R., O’Brien S. (2018). Approaches to Chronic Lymphocytic Leukemia Therapy in the Era of New Agents: The Conundrum of Many Options. Am. Soc. Clin. Oncol. Educ. Book.

[157] Strati P., Jain N., O’Brien S. (2018). Chronic Lymphocytic Leukemia: Diagnosis and Treatment. Mayo Clin. Proc..

[158] Perini G.F., Ribeiro G.N., Pinto Neto J.V., Campos L.T., Hamerschlak N. (2018). BCL-2 as therapeutic target for hematological malignancies. J. Hematol. Oncol..

[159] Seymour J.F., Kipps T.J., Eichhorst B., Hillmen P., D’Rozario J., Assouline S., Owen C., Gerecitano J., Robak T., De la Serna J. (2018). Venetoclax-Rituximab in Relapsed or Refractory Chronic Lymphocytic Leukemia. N. Engl. J. Med..

[160] Koziner B. (2004). Potential therapeutic applications of oblimersen in CLL. Oncology (Williston Park).

[161] Yu B., Mao Y., Bai L.Y., Herman S.E., Wang X., Ramanunni A., Jin Y., Mo X., Cheney C., Chan K.K. (2013). Targeted nanoparticle delivery overcomes off-target immunostimulatory effects of oligonucleotides and improves therapeutic efficacy in chronic lymphocytic leukemia. Blood.

[162] Chiang C.L., Goswami S., Frissora F.W., Xie Z., Yan P.S., Bundschuh R., Walker L.A., Huang X., Mani R., Mo X.M. (2019). ROR1-targeted delivery of miR-29b induces cell cycle arrest and therapeutic benefit in vivo in a CLL mouse model. Blood.

[163] Alfayez M., Kantarjian H., Kadia T., Ravandi-Kashani F., Daver N. (2019). Emerging drug profile: CPX-351 (vyxeos) in AML. Leuk. Lymphoma.

[164] Baron J., Wang E.S. (2018). Gemtuzumab ozogamicin for the treatment of acute myeloid leukemia. Expert Rev. Clin. Pharmacol..

[165] Eghtedar A., Verstovsek S., Estrov Z., Burger J., Cortes J., Bivins C., Faderl S., Ferrajoli A., Borthakur G., George S. (2012). Phase 2 study of the JAK kinase inhibitor ruxolitinib in patients with refractory leukemias, including postmyeloproliferative neoplasm acute myeloid leukemia. Blood.

[166] Winer E.S., Stone R.M. (2019). Novel therapy in Acute myeloid leukemia (AML): Moving toward targeted approaches. Ther. Adv. Hematol..

[167] Massimino M., Stella S., Tirro E., Romano C., Pennisi M.S., Puma A., Manzella L., Zanghi A., Stagno F., Di Raimondo F. (2018). Non ABL-directed inhibitors as alternative treatment strategies for chronic myeloid leukemia. Mol. Cancer.

[168] Pollyea D.A., Tallman M.S., de Botton S., Kantarjian H.M., Collins R., Stein A.S., Frattini M.G., Xu Q., Tosolini A., See W.L. (2019). Enasidenib, an inhibitor of mutant IDH2 proteins, induces durable remissions in older patients with newly diagnosed acute myeloid leukemia. Leukemia.

[169] Phuphanich S., Maria B., Braeckman R., Chamberlain M. (2007). A pharmacokinetic study of intra-CSF administered encapsulated cytarabine (DepoCyt) for the treatment of neoplastic meningitis in patients with leukemia, lymphoma, or solid tumors as part of a phase III study. J. Neurooncol..

[170] Jurczak W., Kroll-Balcerzak R., Giebel S., Machaczka M., Giza A., Ogorka T., Fornagiel S., Rybka J., Wrobel T., Kumiega B. (2015). Liposomal cytarabine in the prophylaxis and treatment of CNS lymphoma: toxicity analysis in a retrospective case series study conducted at Polish Lymphoma Research Group Centers. Med. Oncol..

[171] Chang H.I., Yeh M.K. (2012). Clinical development of liposome-based drugs: Formulation, characterization, and therapeutic efficacy. Int. J. Nanomed..

[172] Levato L., Molica S. (2018). Rituximab in the management of acute lymphoblastic leukemia. Expert Opin. Biol. Ther..

[173] Tobinai K., Klein C., Oya N., Fingerle-Rowson G. (2017). A Review of Obinutuzumab (GA101), a Novel Type II Anti-CD20 Monoclonal Antibody, for the Treatment of Patients with B-Cell Malignancies. Adv. Ther..

[174] Shepard R. (2018). Liposomal Annamycin—A New Generation Anthracycline That Overcomes MDR and Has No Cardiac Toxicity for the Second Line Treatment of R/R AML. Clin. Lymphoma Myeloma Leuk..

[175] Shah N.N., Merchant M.S., Cole D.E., Jayaprakash N., Bernstein D., Delbrook C., Richards K., Widemann B.C., Wayne A.S. (2016). Vincristine Sulfate Liposomes Injection (VSLI, Marqibo(R)): Results From a Phase I Study in Children, Adolescents, and Young Adults With Refractory Solid Tumors or Leukemias. Pediatr. Blood Cancer.

[176] Thomas X., Paubelle E. (2018). Grb2 inhibition: a new potential targeted therapy for myeloid malignancies?. Lancet Haematol..

[177] Ohanian M., Tari Ashizawa A., Garcia-Manero G., Pemmaraju N., Kadia T., Jabbour E., Ravandi F., Borthakur G., Andreeff M., Konopleva M. (2018). Liposomal Grb2 antisense oligodeoxynucleotide (BP1001) in patients with refractory or relapsed haematological malignancies: A single-centre, open-label, dose-escalation, phase 1/1b trial. Lancet Haematol..

[178] Huang H.-Q., Huang Y., Yan G., Yang H., Zhang Q., Yang R., Zhou M., Li Y., Li Y., Liu L. (2019). Safety and Efficacy of Mitoxantrone Hydrochloride Liposome in Patients with Relapsed or Refractory Peripheral T-Cell Lymphoma and Extranodal NK/T-Cell Lymphoma: A Multicenter, Single-Arm, Open-Label, Phase 2 Clinical Trial. Blood.

[179] Yang J., Shi Y., Li C., Gui L., Zhao X., Liu P., Han X., Song Y., Li N., Du P. (2014). Phase I clinical trial of pegylated liposomal mitoxantrone plm60-s: Pharmacokinetics, toxicity and preliminary efficacy. Cancer Chemother. Pharmacol..

[180] Floc’h N., Ashton S., Taylor P., Trueman D., Harris E., Odedra R., Maratea K., Derbyshire N., Caddy J., Jacobs V.N. (2017). Optimizing Therapeutic Effect of Aurora B Inhibition in Acute Myeloid Leukemia with AZD2811 Nanoparticles. Mol. Cancer Ther..

[181] Ashton S., Song Y.H., Nolan J., Cadogan E., Murray J., Odedra R., Foster J., Hall P.A., Low S., Taylor P. (2016). Aurora kinase inhibitor nanoparticles target tumors with favorable therapeutic index in vivo. Sci. Transl. Med..

[182] Song Y.H., Shin E., Wang H., Nolan J., Low S., Parsons D., Zale S., Ashton S., Ashford M., Ali M. (2016). A novel in situ hydrophobic ion paring (HIP) formulation strategy for clinical product selection of a nanoparticle drug delivery system. J. Control. Release.

